# Factors associated with loss to follow-up after point-of-care diabetic retinopathy examination in a teleophthalmology program: A retrospective cohort study

**DOI:** 10.1371/journal.pone.0359305

**Published:** 2026-10-29

**Authors:** Francisco J. Bonilla-Escobar, Sarah Atta, Maria R. Eibel, Joshua S. Wesalo, Jordan T. Chang, Daniel Sánchez-Cano, Andrew Eller, Evan L. Waxman

**Affiliations:** 1 Department of Ophthalmology, University of Pittsburgh, UPMC Vision Institute, Pittsburgh, Pennsylvania, United States of America; 2 Department of Ophthalmology, Grupo de Investigación Visión y Salud Ocular (VISOC), Servicio de Oftalmología, Universidad del Valle, Hospital Universitario del Valle, Evaristo Garcia E.S.E., Cali, Colombia; 3 Fundación Somos Ciencia al Servicio de la Comunidad, Fundación SCISCO / Science to Serve the Community Foundation, SCISCO Foundation, Cali, Valle del Cauca, Colombia; University Hospitals Cleveland, UNITED STATES OF AMERICA

## Abstract

**Objective:**

To identify patient-, clinical-, and system-level factors associated with loss to follow-up after point-of-care diabetic retinopathy examination (DRE).

**Methods:**

We conducted a retrospective cohort study of adults ≥18 years undergoing DREs at 75 primary care clinics in Pennsylvania, USA (2017–2019), restricted to examinations with recommended follow-up. The unit of analysis was the DRE. Electronic medical records were used to obtain six-month follow‑up outcomes and patient-, clinical-, and system-level variables. Loss to follow-up was defined as failure to attend an eye care visit within 180 days of DRE. Examinations with duplicate results, those from patients who died within six months, or from sites where the program was discontinued were excluded. Multivariable mixed-effects logistic regression models with random intercepts for patient and clinic were used. Analyses were conducted in the full cohort and separately for gradable and indeterminate exams (ungradable/no image).

**Results:**

Among 3,121 DREs from 2,959 patients, 32.3% completed follow-up. In the full cohort, higher odds of loss to follow-up were associated with increasing age (adjusted odds ratio [aOR]=1.02, 95% confidence interval [95%CI]=1.00–1.03, p = 0.019), not being married (aOR=1.36, 95%CI = 1.04–1.77, p = 0.03), findings suggestive of age-related macular degeneration (AMD) on DRE (aOR=1.80, 95%CI = 1.14–2.85, p = 0.012), and higher no-show rates (aOR=1.04, 95%CI = 1.02–1.05, p < 0.0001). History of eye disease (aOR=0.50, 95%CI = 0.37–0.69, p < 0.0001) and patient contact within 30 days (e.g., same/next day: aOR=0.37, 95%CI = 0.20–0.67, p = 0.001) were associated with lower odds of loss to follow-up; contact at 31-60 days was not significantly associated. Findings were consistent across full and gradable models; only no-show rate remained significant in the indeterminate model. Socioeconomic variables, comorbidities, and diabetic retinopathy severity were not independently associated with loss to follow-up after adjustment.

**Conclusions:**

Adherence was associated with patient (sociodemographic: age, marital status), clinical (AMD on DRE, prior eye disease), and system-level factors (no-show rates, patient contact within 30 days), supporting targeted, multifactorial interventions.

## Introduction

Diabetic retinopathy (DR) remains a leading cause of preventable vision loss among adults, affecting approximately 9.6 million individuals with diabetes in the United States, of whom 1.84 million have vision-threatening disease (VTD) [1,2]. The American Academy of Ophthalmology recommends annual eye examinations for individuals with diabetes to reduce the risk of vision loss [3]. Despite the effectiveness of early detection and treatment, a substantial care gap persists: only 66% of adults with diabetes undergo annual eye examination [4], and up to 73% of individuals over 40 years of age with DR remain unaware of their diagnosis [5]. Geographic and socioeconomic disparities continue to influence disease burden, and prevalence among adults aged ≥40 years is projected to double by 2050 [2,6].

Point-of-care diabetic retinopathy examination programs (POCDREP) have expanded access to screening; however, follow-up after screening remains inconsistent, with reported rates ranging from 30% to 75% [7–9]. This gap underscores a critical failure point in the care continuum, where successful screening does not guarantee subsequent follow-up care.

Prior studies have identified several sociodemographic and economic factors associated with loss to follow-up after DRE, including younger age [8,10], female sex, urban residence [10], lack of insurance, and greater distance from eye care services [11]. Evidence about ethnicity is inconsistent [12,13]. Clinical factors such as milder DR, absence of comorbidities, diabetes duration of 6–10 years [12], and limited awareness of glycemic control have also been associated with lower adherence [8]. In addition, system-level barriers, including delays in scheduling [12], unclear or insufficient assistance with scheduling [8], ambiguous referral processes, and patients’ perceptions of diabetic retinopathy as untreatable [14], further contribute to missed follow-up. Conversely, emerging technologies like AI-assisted interpretation systems have demonstrated improvements in adherence to recommended care by enhancing the efficiency of result reporting [10].

Previous studies addressing follow-up after diabetic retinopathy examination (DRE) have limitations, including small sample sizes [7,11,13,15], reliance on interviews [14], or being conducted in settings that limit generalizability [10]. Other studies draw from narrowly defined patient groups, such as those seen at one DRE site, by a single provider, or within one insurance plan, which may introduce bias and limit broader applicability [7,8,12–15]. These studies tend to concentrate on specific patient characteristics, often clinical, while overlooking system-related factors that may impact follow-up rates.

To address these limitations, we aimed to identify patient-, clinical-, and system-level factors associated with loss to follow-up after DRE within the University of Pittsburgh/UPMC POCDREP in Western and Central Pennsylvania, United States [9,16].

## Methods

### Study design

This retrospective cohort study evaluated six-month follow-up among adults (≥18 years) undergoing DREs at 75 primary care clinics within the POCDREP network between 2017 and 2019, with analyses restricted to examinations that resulted in a recommendation for follow-up with an ophthalmologist or optometrist. Electronic medical records (EMRs) were used to extract patient-, clinical-, and system-level variables, including the primary outcome of loss to follow-up. Data were accessed from March 1 to August 25, 2020.

The study was conducted in accordance with the Declaration of Helsinki. Ethical approval was obtained from the University of Pittsburgh Institutional Review Board (STUDY19120237, January 28, 2020), which determined the study to be exempt from informed consent as it involved a retrospective analysis of de-identified data.

### Setting

A total of 75 centers across Western and Central Pennsylvania were involved, including 35 equipped with automated retinal cameras and 40 without. Patients from centers without cameras were referred to camera-equipped sites for DRE. Among the 35 equipped clinics, 77.1% (n = 27) were part of the UPMC primary care network, 20% (n = 7) were University of Pittsburgh Physician Offices, and one was a free clinic (2.9%).

Clinic staff used either the Centervue DRS (iCare, Revenio Group) or Topcon TRC-NW400 digital retinal camera to capture macula-centered, 45-degree images of each eye. Images were stored and transmitted through the Epic® system, then graded by two ophthalmologists using a standardized protocol based on the International Clinical Diabetic Retinopathy Severity Scale [17]. Disagreements were resolved by consensus. Standardized reports were generated and communicated to ordering physicians via the EMR or secure email.

Based on imaging interpretation, follow-up was recommended in the presence of clinically significant findings, including moderate or worse DR or DR with macular exudates [17,18], other ocular pathologies such as age-related macular degeneration (AMD), suspected glaucoma, branch retinal vein occlusion (BRVO), among others, as well as indeterminate examinations (e.g., non-gradable or missing images). Follow-up recommendations were based on screening results rather than predefined time intervals.

### Participants

A total of 12,444 DREs were performed between January 1, 2017 and December 31, 2019. The unit of analysis was the DRE. We included exams from adults (≥18 years) performed within the POCDREP during this time period that resulted in a recommendation for follow-up with an eye care provider (n = 3,738).

We excluded duplicate exams with identical findings (n = 369), exams from patients who died within 6 months of the index DRE (n = 1), and exams from sites where the program became inactive shortly after implementation (n = 247). The final analytic sample included 3,121 exams (25.1% of all DREs performed during the study period).

### Variables

The dependent variable was loss to follow-up, defined as failure to attend an eye care visit (optometrist or ophthalmologist) within 180 days after the index DRE, regardless of whether the visit addressed diabetic retinopathy or other ocular conditions. Follow-up included eye care encounters within the UPMC system as well as external eye care encounters documented in the UPMC EMR. External encounters were identified when documentation from outside providers, such as consultation notes or examination reports, had been entered or uploaded into the patient’s record. The study did not independently query outside health systems or eye care practices; therefore, external encounters that were not documented in the UPMC EMR could not be identified and may have been classified as loss to follow-up.

Independent variables included patient-level sociodemographic and economic factors, clinical factors, and system-level factors.

### Patient-level factors

Sociodemographic and economic characteristics included age, sex, race/ethnicity, marital status, insurance type, and neighborhood poverty level. Marital status was categorized as ‘Married’ (including those legally married or in a stable partnership), and ‘Not Married’ (including single, divorced, legally separated, widowed, or unspecified). Insurance coverage was grouped into Medicaid, Medicare, private/commercial insurance, dual coverage, and uninsured or Veterans Affairs (VA). Zip codes were used to estimate socioeconomic status as described elsewhere [19–21]. Neighborhood poverty was categorized into quintiles, from the lowest to highest: < 10% (Q1), 10–19.9% (Q2), 20–29.9% (Q3), 30–39.9% (Q4), ≥ 40% (Q5), based on US Census Bureau definitions of poverty areas.

### Clinical factors

Clinical variables included diabetes type, HbA1c levels, body mass index (BMI), insulin use, and smoking status at the time of the first DRE. We used the EMR problem list to derive the total number of medical conditions (numeric count) and categorical variables indicating the presence or absence of conditions grouped by system or domain, referred to collectively as comorbidities. We also included the number of emergency room (ER) visits in the six months before and after the DRE.

DRE findings included DR severity and the presence of suspected VTDR, defined as more than mild DR or any DR with macular exudates [17,18]. Other ocular findings, such as AMD, BRVO, glaucoma, and hypertensive retinopathy, among others, were recorded based on image interpretation.

### System-level factors

We assessed key aspects of the POCDREP process, including timing of care delivery and follow-up communication. To evaluate workflow variability, we used recorded dates of DRE, interpretation, and patient contact to calculate two timing variables: (1) days from DRE to interpretation, and (2) days from DRE to patient contact. The latter was highly skewed with a long right tail. Days from exam to contact by primary care provider (PCP) or the Eye Center were recategorized as follows: Not contacted, Same or next day, 2–3 days, 4–5 days, 6–7 days, 8–14 days, 15–30 days, 31–60 days, and >60 days.

Communication-related variables included the presence of a referral to eye care, documentation of patient contact by the primary care site or eye center to relay exam results, and the method of contact. Contact method was classified into seven mutually exclusive categories based on communication type, with patients assigned to the most resource-intensive method. For modelling, these were collapsed into four groups: not contacted, low-tech asynchronous (letter or email), phone-based (voicemail or telephone), and in-person interaction.

Image quality for each exam was classified as gradable or indeterminate. Exams were considered gradable if at least one image allowed assessment and a diagnostic impression could be made, and indeterminate if all images were ungradable, missing, or no diagnosis could be made. Additional variables included referring clinic type (family medicine, internal medicine, combined, or endocrinology) and the patient’s overall rate of missed appointments (defined as no-shows across all medical visits).

### Data sources

Data were obtained from automated POCDREP reports and the Epic® EMR. Patient-level (sociodemographic and economic) variables were extracted from program reports, while clinical and system-level variables were obtained from the EMR.

### Data collection strategy

The EMR was reviewed to identify follow-up encounters with eye care providers (UPMC Eye Center or community providers) using a standardized procedure [9]. The search was conducted twice by different members of the research team to ensure accuracy. Reviewers searched for encounters within the UPMC Eye Center and for documentation of care provided by community or other external eye care provider. For external eye care, reviewers examined clinical documentation and uploaded records, including the Media section of the EMR. External encounters for which no documentation was available in the UPMC records could not be captured. Discrepancies were found in 7.7% (239/3,121) of the revised charts. These included misclassifying patients as not having follow-up when they did (n = 69), as having follow-up when they did not (n = 50), or recording an incorrect follow-up date (n = 58), among others. A team member with two years of experience in the POCDREP adjudicated discrepancies based on a final EMR review.

### Statistical analysis

#### Univariate and bivariate analysis.

Continuous variables were described using measures of central tendency and dispersion; categorical variables, using frequencies and percentages. We conducted bivariate analyses to compare patients with and without follow-up, using t-tests or Wilcoxon rank-sum tests for continuous variables depending on distributional assumptions, and Chi-square tests for categorical variables.

#### Multivariable analysis.

To evaluate factors independently associated with loss to follow-up after DRE, we developed three complementary multivariable mixed-effects logistic regression models incorporating patient-, clinical-, and system-level variables. All models included random intercepts for patient and clinic sites to account for clustering of examinations within individuals and sites.

Model A was used to estimate the overall association between image gradability and loss to follow-up, adjusting for patient, clinical, and system-level factors. To account for the fact that clinical retinal information is only available when images are gradable, we then stratified the analysis by gradability. Model B examined predictors of loss to follow-up among examinations with interpretable images, where clinical information is available to potentially influence follow-up behavior. Model C examined predictors of loss to follow-up among examinations without interpretable images, where follow-up is expected to depend primarily on patient and system-level factors rather than clinical findings. Models B and C included 2,536 and 400 examinations, respectively, after excluding observations with missing model covariates.

Variable selection followed a two-step approach. First, we included variables identified in the literature as relevant to follow-up outcomes, such as age [8,10], sex [10], race/ethnicity [12,13], insurance coverage [13], severity of retinopathy, presence of comorbidities [12], and whether the patient received assistance scheduling the follow-up appointment [8]. This last factor was represented by a proxy variable indicating whether the patient was contacted by their PCP or the Eye Center to communicate exam results or facilitate scheduling follow-up care. To avoid collinearity between type of contact and time from DRE to contact, the reference category for the latter was recategorized to include cases with >60 days between DRE and contact in the no contact group. Continuous variables (age, BMI, number of problems, and no-show rate) were mean-centered to facilitate model interpretation. Second, we performed simple logistic regressions clustered by clinic site and retained variables with p < 0.20 for inclusion in the multivariable models. Multicollinearity was assessed prior to mixed-effects modeling using uncentered variance inflation factors (VIF) derived from single-level logistic regression models; all variables had VIF < 10.

All models used mixed-effects logistic regression with random intercepts for patient and clinic site to account for clustering of examinations within individuals and sites. The intraclass correlation coefficient (ICC) was used to quantify the proportion of variance attributable to clustering; although the ICC for site was small, random effects were retained to ensure robust standard errors. Model fit was assessed using the Akaike and Bayesian information criteria (AIC and BIC). The Wald test was used to test the significance of interactions. Statistical significance was set at p-value <0.05, and all analyses were conducted using Stata 19 (StataCorp, TX)®.

## Results

### Univariate analysis

A total of 3,121 DREs from 2,959 unique patients who were recommended for ophthalmic follow-up were included in the analysis. Within six months (180 days) following the exam date, 32.3% (1,009) of DREs were followed by a completed eye care visit.

The mean age was 62.8 years (standard deviation [SD]=13.5), 54.9% of patients were male, 63.0% were White, and 55.5% were not married. Medicare was the most common insurance type (42.1%), and most patients resided in ZIP codes with <20% of residents living below the federal poverty level.

The median HbA1c was 7.1% (interquartile range [IQR]=6.3–8.6), and the median BMI was 32.1 kg/m^2^ (IQR: 27.7–37.3). Most patients had type 2 diabetes (91.4%), and 42.1% were using insulin. Comorbidities were common, including cardiovascular disease (82.1%), obesity (37.0%), mental illness (31.8%), kidney disease (24.3%), lung disease (22.4%), pain conditions (45.9%), substance use disorders (19.6%), cancer (16.0%), neurological disorders (10.6%), mobility issues (42.1%), cognitive impairment (3.8%), and hearing conditions (6.5%). Eye disease was documented in 29.4%, and diabetes-related complications in 72.1%. The median number of emergency room visits within six months before or after the DRE was 0 (IQR: 0–1).

Most DREs were gradable (86.6%); among gradable examinations, 70.9% showed no DR and 24.6% were classified as VTDR. Additional findings included suspected glaucoma (23.8%), AMD (9%), hypertensive retinopathy (4.5%) and BRVO (2.3%).

### Bivariate analysis

#### Patient-level factors.

Marital status was significantly associated with follow-up, with higher completion among married individuals compared to those not married (35.3% vs. 29.9%; p = 0.004) as shown in Table 1. No other sociodemographic or economic variables were significantly associated with follow-up status (all p > 0.05).

**Table 1:** Bivariate comparison of patient-level factors by follow-up status among diabetic retinopathy exams (N = 3,121 exams from 2,959 patients).

Characteristic	Total (*Column %*)	Completed Follow-Up (n = 1,009) (*Row %*)	Loss to Follow-Up (n = 2,112) (*Row %*)	p-value
Age, mean (SD)	62.8 (13.5)	63.1 (13.2)	62.6 (13.6)	0.33
Sex, n (%)				0.89
Female	1,407 (45.1)	453 (32.2)	954 (67.8)	
Male	1,714 (54.9)	556 (32.4)	1,158 (67.6)	
Race, n (%)				0.53
White	1,967 (63.0)	631 (32.1)	1,336 (67.9)	
Black	1,012 (32.4)	326 (32.2)	686 (67.8)	
Other	142 (4.5)	52 (36.6)	90 (63.4)	
Marital status, n (%)				0.004
Not married	1,733 (55.5)	518 (29.9)	1,215 (70.1)	
Married	1,377 (44.1)	486 (35.3)	891 (64.7)	
*Missing*	11 (0.4)	5 (45.5)	6 (54.5)	
Insurance, n (%)				0.33
Medicare	1,314 (42.1)	438 (33.3)	876 (66.7)	
Commercial	846 (27.1)	279 (33.0)	567 (67.0)	
Medicaid	455 (14.6)	130 (28.6)	325 (71.4)	
Dual	295 (9.5)	101 (34.2)	194 (65.8)	
Uninsured	34 (1.1)	13 (38.2)	21 (61.8)	
Veterans Affairs	11 (0.4)	3 (27.3)	8 (72.7)	
*Missing*	166 (5.3)	45 (27.1)	121 (72.9)	
Poverty level, n (%)				0.85
<10% (Q1)	1,935 (62.0)	620 (32.0)	1,315 (68.0)	
10-19.9% (Q2)	881 (28.2)	287 (32.6)	594 (67.4)	
20-29.9% (Q3)	138 (4.4)	43 (31.2)	95 (68.8)	
30-39.9% (Q4)	135 (4.3)	46 (34.1)	89 (65.9)	
*Missing*	32 (1.0)	13 (40.6)	19 (59.4)	

Legend: Values are presented as mean (SD) for continuous variables and n (%) for categorical variables. Percentages in the total column represent column percentages, whereas percentages in the follow-up columns represent row percentages. Bivariate comparisons were performed using t-tests for continuous variables and Chi-square tests for categorical variables. Q: quintile.

### Clinical factors

Current smoking was associated with lower follow-up (p = 0.022). The median number of medical conditions was higher among exams with completed follow-up compared with those without follow-up (13 vs 11; p < 0.001). The presence of mobility-related conditions (p = 0.021), history of eye disease (p < 0.001), and diabetes-related complications (p = 0.012) were associated with higher follow-up completion rates (Table 2).

**Table 2:** Bivariate comparison of clinical factors by follow-up status among diabetic retinopathy exams (N = 3,121 exams from 2,959 patients).

Characteristic	Total (*Column %*)	Completed Follow-Up (n = 1,009) (*Row %*)	Loss to Follow-Up (n = 2,112) (*Row %*)	p-value
Diabetes type				0.10
Type 1	269 (8.6)	99 (36.8)	170 (63.2)	
Type 2	2,852 (91.4)	910 (31.9)	1,942 (68.1)	
Insulin use				0.12
No	1,807 (57.9)	564 (31.2)	1,243 (68.8)	
Yes	1,314 (42.1)	445 (33.9)	869 (66.1)	
Smoking status, n (%)				0.022
Never smoked	1,138 (36.5)	372 (32.7)	766 (67.3)	
Former smoker	1,326 (42.5)	453 (34.2)	873 (65.8)	
Current smoker	653 (20.9)	183 (28.0)	470 (72.0)	
*Missing*	4 (0.1)	1 (25.0)	3 (75.0)	
Number of medical conditions, median (IQR)	12 (8–18), n = 3,120	13 (8-18)	11 (7–17), n = 2,111	<0.001
Comorbidities, n (%)				
Mobility-related conditions				0.021
No	1,806 (57.9)	554 (30.7)	1,252 (69.3)	
Yes	1,315 (42.1)	455 (34.6)	860 (65.4)	
History of eye disease				<0.001
No	2,202 (70.6)	636 (28.9)	1,566 (71.1)	
Yes	919 (29.4)	373 (40.6)	546 (59.4)	
Diabetes-related complications				0.012
No	870 (27.9)	252 (29.0)	618 (71.0)	
Yes	2,251 (72.1)	757 (33.6)	1,494 (66.4)	
Highest level of DR per exam, n (%)				0.008
No DR	1,916 (61.4)	606 (31.6)	1,310 (68.4)	
Mild	123 (3.9)	35 (28.5)	88 (71.5)	
Moderate	269 (8.6)	94 (34.9)	175 (65.1)	
Severe	62 (2.0)	29 (46.8)	33 (53.2)	
Proliferative	82 (2.6)	35 (42.7)	47 (57.3)	
Macular exudate	251 (8.0)	92 (36.7)	159 (63.3)	
*Missing*	418 (13.4)	118 (28.2)	300 (71.8)	
VTDR, n (%)				0.002
Absent	2,039 (65.3)	641 (31.4)	1,398 (68.6)	
Present	664 (21.3)	250 (37.7)	414 (62.3)	
*Not applicable* ^a^	418 (13.4)	118 (28.2)	300 (71.8)	
AMD, n (%)				0.003
Absent	2,418 (77.5)	818 (33.8)	1,600 (66.2)	
Present	281 (9.0)	72 (25.6)	209 (74.4)	
*Not applicable* ^a^	422 (13.5)	119 (28.2)	303 (71.8)	

Legend: Values are presented as median (IQR) for continuous variables and n (%) for categorical variables. Percentages in the total column represent column percentages, whereas percentages in the follow-up columns represent row percentages. Non-missing n is reported where applicable for continuous variables. Bivariate comparisons were performed using the Wilcoxon rank-sum test for continuous variables and Chi-square tests for categorical variables. VTDR: vision-threatening diabetic retinopathy. IQR: interquartile range. a. Indeterminate images.

Follow-up rates varied by DR severity (p = 0.008), with higher completion among exams with more advanced disease, including severe (46.8%), proliferative (42.7%), and macular exudate (36.7%), compared with no DR (31.6%) and mild DR (28.5%). Although overall follow-up was low among patients with VTDR, they were significantly more likely to attend follow-up than those without VTDR (37.7% vs 31.4%; p = 0.002). In contrast, examinations with findings suggestive of AMD had lower follow-up completion rates than those without AMD (25.6% vs 33.8%; p = 0.006; excluding indeterminate AMD assessments).

Other clinical variables, including ER visits, image gradability, glaucoma suspicion, hypertensive retinopathy, BRVO, and other comorbidities (e.g., mental health conditions, cognitive impairment, neurological disorders, etc.) were not significantly associated with follow-up status (all p > 0.05).

### System-level factors

Higher no-show rates were associated with lower follow-up (p < 0.001). Documented patient contact was associated with higher follow-up (36.8% vs 22.4% among those not contacted; p < 0.001), and longer delays between DRE and contact were associated with lower follow-up (p < 0.001).

Follow-up differed by clinic specialty (p = 0.01), with the highest completion in internal medicine clinics (36.2%). No significant differences were observed for time from DRE to interpretation (p = 0.43), image gradability (p = 0.054), or referral to eye care (p = 0.15) (**Table 3**).

**Table 3:** Bivariate comparison of system-level factors by follow-up status among diabetic retinopathy exams (N = 3,121 exams from 2,959 patients).

Characteristic	Total (n = 3,121) (*Column %*)	Completed Follow-Up (n = 1,009) (*Row %*)	Loss to Follow-Up (n = 2,112) (*Row %*)	p-value
Days from DRE to patient contact, median (IQR)	5 (2–15), n = 2,604	5 (2–13), n = 893	5 (2–16), n = 1,711	<0.001
Time from DRE to patient contact, n (%)				<0.001
No recorded contact date	517 (16.6)	116 (22.4)	401 (77.6)	
Same or next day	526 (16.9)	198 (37.6)	328 (62.4)	
2-3 days	473 (15.2)	161 (34.0)	312 (66.0)	
4-5 days	367 (11.8)	131 (35.7)	236 (64.3)	
6-7 days	257 (8.2)	100 (38.9)	157 (61.1)	
8-14 days	326 (10.4)	110 (33.7)	216 (66.3)	
15-30 days	308 (9.9)	108 (35.1)	200 (64.9)	
31-60 days	154 (4.9)	42 (27.3)	112 (72.7)	
>60 days	193 (6.2)	43 (22.3)	150 (77.7)	
Method of Contact, n (%)				<0.001
No documented contact method	519 (16.6)	116 (22.4)	403 (77.6)	
Letter only	204 (6.5)	39 (19.1)	165 (80.9)	
Email only	187 (6.0)	62 (33.2)	125 (66.8)	
Voicemail only	306 (9.8)	101 (33.0)	205 (67.0)	
Telephone only	1,453 (46.6)	542 (37.3)	911 (62.7)	
Multimodal (no in-person)	198 (6.3)	65 (32.8)	133 (67.2)	
In-person involved	254 (8.1)	84 (33.1)	170 (66.9)	
Image gradability, n (%)				0.054
No	418 (13.4)	118 (28.2)	300 (71.8)	
Yes	2,703 (86.6)	891 (33.0)	1,812 (67.0)	
Clinic Specialty, n (%)				0.01
Family medicine	1,636 (52.4)	492 (30.1)	1,144 (69.9)	
Internal medicine	1,040 (33.3)	377 (36.2)	663 (63.7)	
Combined practice (family and internal medicine)	266 (8.5)	82 (30.8)	184 (69.2)	
Endocrinology	179 (5.7)	58 (32.4)	121 (67.6)	
No-show rate, median (IQR)	10 (4–21), n = 3,120	9 (3–18), n = 1,009	10 (4–22), n = 2,111	<0.001
No show rate category, n (%)				
0-4%	894 (28.6)	320 (35.8)	574 (64.2)	
5-10%	754 (24.2)	258 (34.2)	496 (65.8)	
11-21%	738 (23.6)	242 (32.8)	496 (67.2)	
22-86%	734 (23.5)	189 (25.7)	545 (74.3)	
*Missing*	1 (0.03)	0 (0.0)	1 (100.0)	

Legend: Values are presented as median (IQR) for continuous variables and n (%) for categorical variables. Percentages in the total column represent column percentages, whereas percentages in the follow-up columns represent row percentages. Non-missing n is reported where applicable for continuous variables. No-show rate categories represent quartiles of the no-show rate distribution in the study population. Each examination may yield multiple findings for a patient. Bivariate comparisons were performed using the Wilcoxon rank-sum test for continuous variables and Chi-square tests for categorical variables.

### Multivariable analysis

#### Associated factors with loss to follow-up.

In Model A (all DREs), indeterminate images were not significantly associated with loss to follow-up (aOR=1.39, 95%CI = 0.96–2.02, p = 0.079). Older age (aOR=1.016 per year, 95%CI = 1.00–1.03, p = 0.019), not being married (aOR=1.36, 95%CI = 1.04–1.77, p = 0.025), higher no-show rate (aOR=1.04 per percentage point, 95%CI = 1.02–1.05, p < 0.001), and AMD detected on DRE (aOR=1.80, 95%CI = 1.14–2.85, p = 0.012) were associated with higher odds of loss to follow-up. A history of eye disease was associated with lower odds (aOR=0.50, 95%CI = 0.37–0.69, p < 0.001).

Shorter intervals from DRE to patient contact were associated with lower odds of loss to follow-up compared with no contact (Fig 1), including same or next day (aOR=0.37, 95%CI = 0.20–0.67, p = 0.001), 2−3 days (aOR=0.43, 95%CI = 0.23–0.80, p = 0.007), 4−5 days (aOR=0.42, 95%CI = 0.22–0.79, p = 0.007), 6−7 days (aOR=0.33, 95%CI = 0.17–0.66, p = 0.002), 8−14 days (aOR=0.43, 95%CI = 0.22–0.82, p = 0.010), and 15−30 days (aOR=0.37, 95%CI = 0.19–0.71, p = 0.003), with no significant associations observed beyond 30 days (Table 4).

**Table 4:** Factors associated with loss to follow-up after diabetic retinopathy examination in a point-of-care screening program in the US – model A (Full cohort).

Variables	Unadjusted OR (95% CI)	Adjusted OR (95% CI)	p-value
Age (per year, centered)	0.996 (0.988-1.005)	1.016 (1.003-1.029)	0.019
Sex			
Female	(ref)		
Male	0.960 (0.773-1.194)	0.923 (0.722-1.181)	0.526
Race			
Other	(ref)		
Black	1.352 (0.787-2.321)	1.091 (0.595-2.003)	0.778
White	1.272 (0.754-2.144)	1.450 (0.801-2.624)	0.220
Marital status			
Married	(ref)		
Not married	1.456 (1.150-1.843)	1.358 (1.040-1.773)	0.025
Insurance			
Commercial	(ref)		
Dual	0.932 (0.617-1.408)	0.709 (0.439-1.145)	0.159
Medicaid	1.355 (0.935-1.965)	1.110 (0.743-1.658)	0.612
Medicare	0.971 (0.743-1.270)	0.770 (0.547-1.084)	0.134
Veterans Affairs/Uninsured	0.930 (0.343-2.522)	0.746 (0.245-2.278)	0.607
BMI (kg/m², centered)	1.015 (1.001-1.030)	1.015 (0.999-1.032)	0.061
Number of medical conditions (centered)	0.980 (0.966-0.993)	0.985 (0.968-1.002)	0.079
Insulin use			
No	(ref)		
Yes	0.838 (0.669-1.049)	0.885 (0.681-1.151)	0.363
Smoking status			
Never smoked	(ref)		
Former smoker	1.089 (0.858-1.383)	1.072 (0.819-1.403)	0.612
Current smoker	1.443 (1.070-1.944)	1.229 (0.880-1.716)	0.227
Diabetes-related complications			
No	(ref)		
Yes	0.721 (0.558-0.931)	0.800 (0.604-1.060)	0.121
History of eye disease			
No	(ref)		
Yes	0.467 (0.348-0.627)	0.503 (0.367-0.689)	<0.001
Mobility-related conditions			
No	(ref)		
Yes	0.797 (0.638-0.997)	0.922 (0.705-1.207)	0.557
VTDR on DRE			
No	(ref)		
Yes	0.641 (0.487-0.845)	0.864 (0.629-1.188)	0.368
AMD on DRE			
No	(ref)		
Yes	1.634 (1.094-2.442)	1.803 (1.141-2.850)	0.012
No-show rate (per percentage point, centered)	1.027 (1.015-1.038)	1.036 (1.021-1.051)	<0.001
Image gradability			
Gradable	(ref)		
Indeterminate	1.350 (0.968-1.882)	1.394 (0.962-2.021)	0.079
Referral to eye care			
No	(ref)		
Yes	0.815 (0.643-1.033)	0.903 (0.697-1.170)	0.440
Time from DRE to patient contact			
No documented contact date or contact after 60 days	(ref)		
Same or next day	0.354 (0.235-0.533)	0.367 (0.200-0.673)	0.001
2-3 days	0.427 (0.283-0.643)	0.427 (0.229-0.796)	0.007
4-5 days	0.380 (0.244-0.593)	0.417 (0.220-0.792)	0.007
6-7 days	0.317 (0.192-0.524)	0.329 (0.166-0.655)	0.002
8-14 days	0.429 (0.275-0.670)	0.429 (0.224-0.819)	0.010
15-30 days	0.371 (0.230-0.597)	0.365 (0.187-0.710)	0.003
31-60 days	0.653 (0.373-1.143)	0.697 (0.332-1.463)	0.340
Contact method			
No documented contact method	(ref)		
Low-tech asynchronous	0.509 (0.337-0.769)	1.339 (0.708-2.534)	0.369
Phone-based	0.319 (0.210-0.485)	0.822 (0.444-1.522)	0.533
In-person	0.385 (0.229-0.646)	1.025 (0.531-1.979)	0.941
Clinic specialty			
Combined practice (family and internal medicine)	(ref)		
Endocrinology	1.021 (0.468-2.228)	1.060 (0.465-2.417)	0.890
Family Medicine	1.039 (0.633-1.705)	1.199 (0.714-2.015)	0.493
Internal Medicine	0.696 (0.413-1.172)	0.694 (0.397-1.213)	0.200

Legend: CI: Confidence interval; BMI: Body Mass Index; DRE: Diabetic Retinopathy Examination; VTDR: Vision-threatening diabetic retinopathy; AMD: Age related Macular Degeneration. Reference categories (ref) are indicated for each variable. Continuous variables were mean-centered.

**Fig 1:**
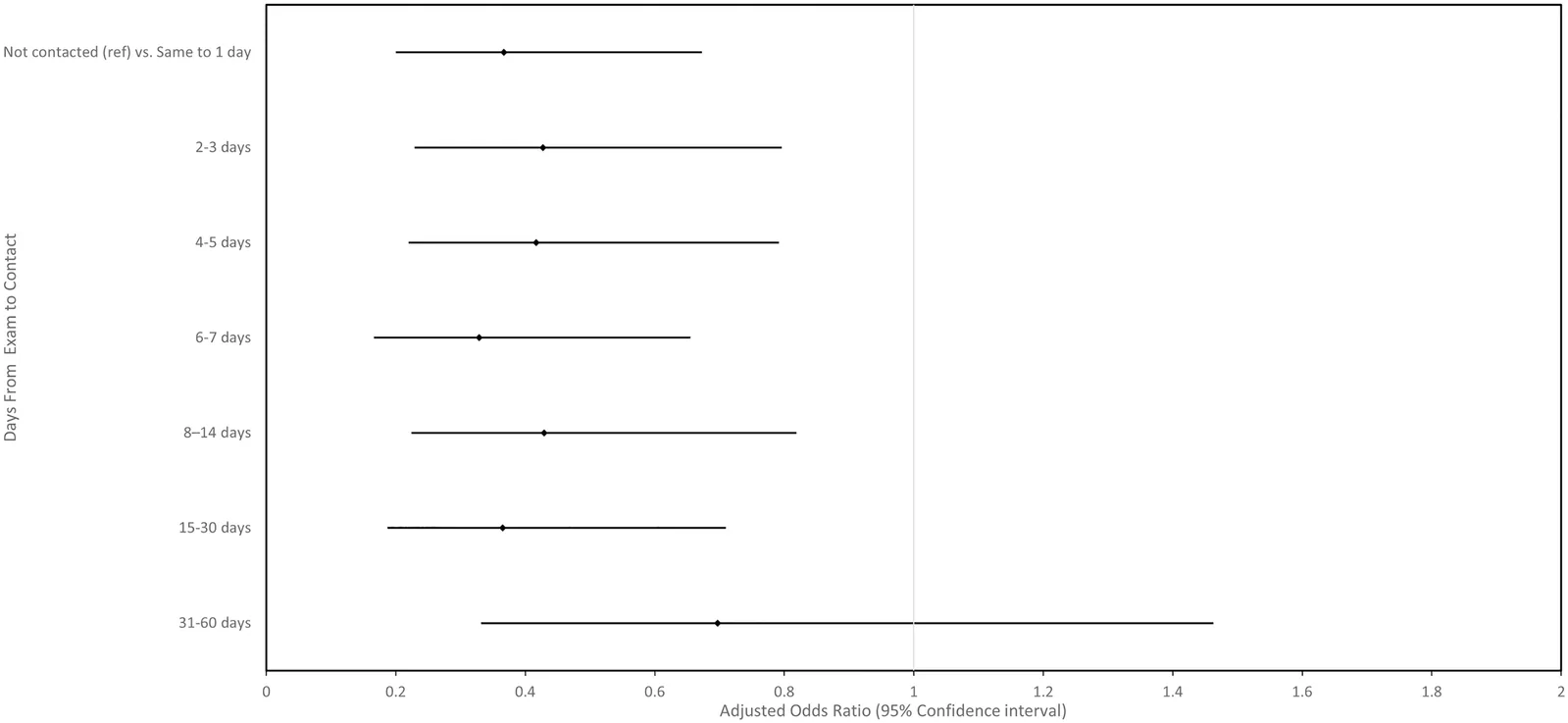
Adjusted odds of loss to follow-up by time from diabetic retinopathy examination to patient contact in a point-of-care program in the United States. Adjusted odds ratios (aOR) and 95% confidence intervals for loss to follow-up by time from diabetic retinopathy examination (DRE) to patient contact, estimated from a mixed-effects logistic regression model with random intercepts for patient and clinic. The reference category includes no recorded contact or contact after 60 days. Contact within 1–30 days was associated with lower odds of loss to follow-up; no significant associations were observed for intervals beyond 30 days.

Model B (S1 Table), restricted to gradable DREs (n = 2,536), showed that AMD remained associated with higher odds of loss to follow-up (aOR=1.75, 95%CI = 1.12–2.73, p = 0.014). Older age (aOR=1.02 per year, 95%CI = 1.00–1.03, p = 0.025), not being married (aOR=1.42, 95% CI = 1.07–1.87, p = 0.015), and higher no-show rate (aOR=1.03 per percentage point, 95%CI = 1.02–1.05, p < 0.001) were also associated with higher odds, whereas a history of eye disease remained protective (aOR=0.48, 95%CI = 0.34–0.67, p < 0.001). Shorter DRE-to-contact intervals were associated with lower odds of loss to follow-up.

Model C (S2 Table), restricted to indeterminate DREs (n = 400), showed that no-show rate was associated with higher odds of loss to-follow-up (aOR=1.04 per percentage point, 95%CI = 1.01–1.07, p = 0.018). No other variables were significantly associated.

Across models A and B, older age, not being married, and higher no-show rates were associated with higher odds of loss to follow-up, whereas only no-show rate remained significant in Model C. A history of eye disease was associated with lower odds in Model A and B but not in Model C. AMD remained a significant predictor in Models A and B but was not assessable in Model C. Shorter DRE-to-contact intervals were associated with lower odds of loss to follow-up in Models A and B, with similar but non-significant patterns observed in Model C.

We tested multiple interaction terms to evaluate whether the effects of key predictors varied across patient subgroups (S3 Table). Although some interactions reached nominal significance (eye diseases × referral to ophthalmology, p = 0.040; diabetic complications × BMI, p = 0.039; contact method × referral to ophthalmology, p = 0.052; clinic specialty × no-show rate, p = 0.060), the likelihood ratio test comparing the full and reduced models did not support their inclusion (LR χ^2^(3) = 6.00, p = 0.111), and model fit metrics (AIC, BIC) favored the main-effects model. Therefore, the final models excluded interaction terms.

Model fit was assessed using AIC (3575.95) and BIC (3803.37). The intraclass correlation coefficient indicated minimal clustering at the site level (ICC = 0.010, 95%CI = 0.002–0.047) and moderate clustering at the patient level (ICC = 0.455, 95%CI = 0.253–0.673). The likelihood ratio test comparing the mixed-effects model with a standard logistic regression was significant (χ^2^(2)=15.85, p = 0.0004), supporting inclusion of random effects.

## Discussion

This large retrospective cohort study of more than 3,100 diabetic retinopathy examinations across a primary care network found that loss to follow-up after a recommended eye care visit was associated with patient-level, clinical, and system-level factors. Older age, not being married, higher prior no-show rate, and AMD detected on DRE were associated with higher odds of loss to follow-up, whereas a documented history of eye disease was associated with lower odds. Timely patient contact after DRE was one of the strongest modifiable factors: contact within 30 days was associated with lower odds of loss to follow-up, while later contact was not significantly associated with improved adherence. These findings suggest that follow-up after point-of-care retinal screening depends not only on patient characteristics or retinal findings, but also on the efficiency of communication workflows that connect screening results to completed eye care.

Findings were consistent across the full cohort (Model A) and gradable DRE models (Model B), whereas in indeterminate examinations (Model C), only no-show rate remained independently associated with loss to follow-up. This suggests that when retinal findings are unavailable, prior healthcare-use behavior may be more informative than clinical retinal characteristics.

We identified key variables that help predict individuals at higher risk of missing follow-up after a DRE recommendation. Recognizing these factors enables healthcare providers to implement targeted interventions, enhancing patient engagement and continuity of care (Fig 2).

**Fig 2:**
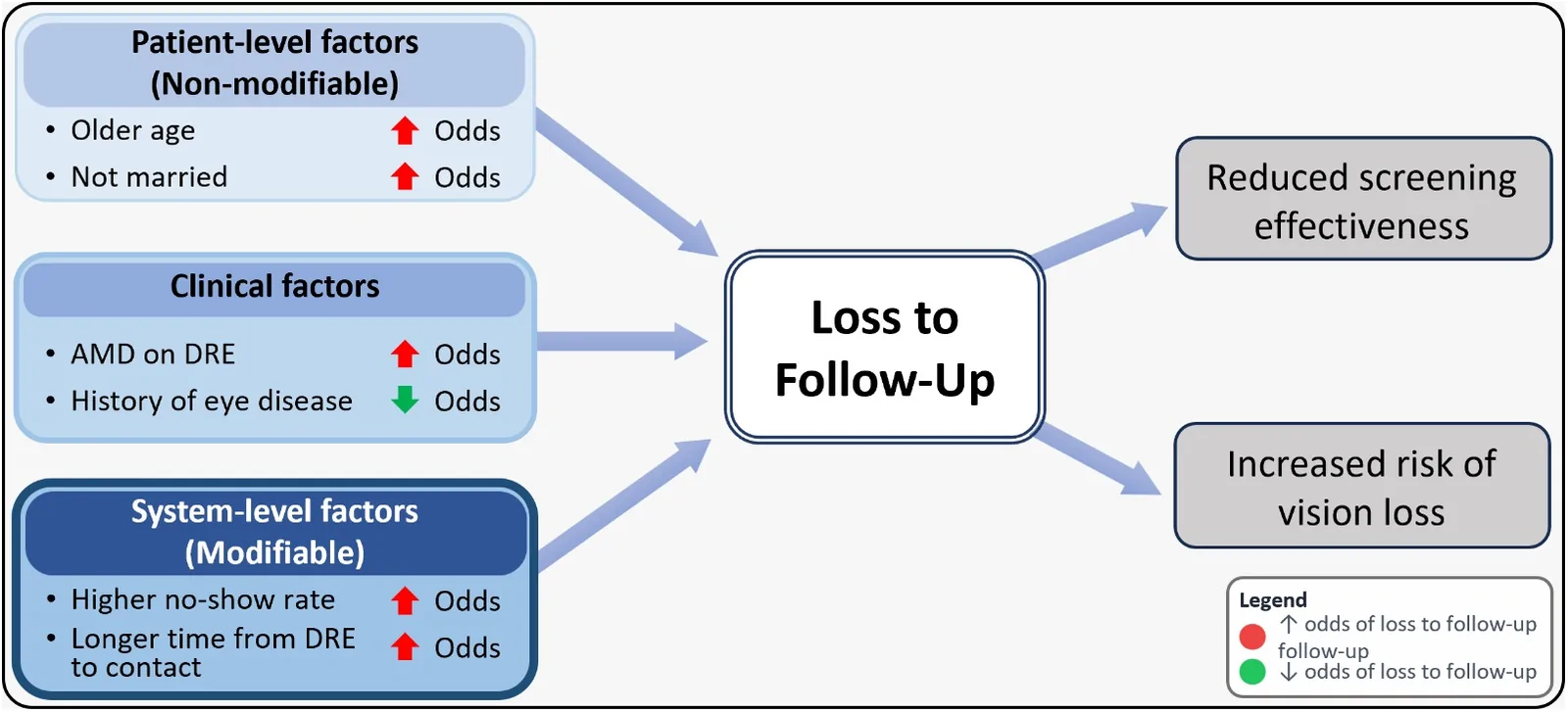
Conceptual framework of factors associated with loss to follow-up after diabetic retinopathy examination in a point-of-care diabetic retinopathy screening program. This diagram summarizes patient-, clinical-, and system-level factors associated with loss to follow-up after diabetic retinopathy examination (DRE) based on multivariable analyses. Red arrows indicate higher odds and green arrows indicate lower odds of loss to follow-up. Patient-level factors (older age, not being married), clinical factors (age-related macular degeneration and history of eye disease [protective]), and system-level factors (higher no-show rate and longer time from DRE to patient contact) influence the odds of loss to follow-up (a linear increase in odds with longer delays was not established). System-level factors are modifiable and represent potential targets for intervention.

### Patient-level factors associated with loss to follow-up

Other studies have described younger age [8,10], sex (female), site of residence [10], distance to the eye centers [11], and Latino ethnicity [12,13], as associated factors with loss to follow-up. We adjusted for most variables except distance and ethnicity. Geographic variables were not included due to identifiability concerns, and the proportion of Latino patients in our cohort was low (2.5%) [22], limiting statistical power to evaluate ethnic differences. Prior studies showing ethnic associations were biased toward Latino populations, as they comprised 65% and 72% Latinos [12,13], which exceeded the actual Latino population in their respective study areas (Riverside, CA: 52% Latino; Durham, NC: 13.9% Latino). As such, differences in population structure and sampling may partly explain discrepancies in findings across studies.

Our study demonstrated that not having a partner/being single is associated with higher odds of loss to follow-up in both bivariate analyses and multivariable Models A and B. This aligns with public health evidence showing that marital status influences health behaviors, improving follow-up, treatment adherence, prognosis, survival rates, and overall health outcomes [23,24].

Older age also remained a modest but statistically significant predictor of loss to follow-up in multivariable Models A and B, linking each additional year of age to a 2% increase in the odds of being lost to follow-up, though conflicting results emerged from other studies. For instance, research in Alabama with predominantly uninsured and Black patients and another study in a Federally Qualified Health Center (FQHC) serving a predominantly Latino population found a positive correlation between age and follow-up [8,13], while a single-center study in California, primarily involving Latinos, did not confirm this association [12].

Age likely reflects underlying barriers rather than acting as a direct causal factor. Older individuals may have previously received a diagnosis of eye disease and therefore perceive follow-up as unnecessary, or that age-related barriers, such as reduced mobility, cognitive decline, or competing comorbidities, may contribute to lower follow-up rates [25]. Social factors may also contribute; older adults are more likely to be unmarried or have limited support networks [26], which have been associated with reduced healthcare engagement.

We assessed socioeconomic status using census-derived ZIP code-level neighborhood poverty rates as a proxy [19–21], rather than individual-level income. Unlike previous studies that relied on a Neighborhood Deprivation Index (NDI) [27,28] or insurance type [12,29], our approach used the proportion of residents living below the federal poverty threshold. Murchison et al. and Peavey et al. found a correlation between their poverty surrogate and compliance with dilated fundus examinations conducted by ophthalmologists [27,29]. In contrast, we did not observe an association between neighborhood poverty and loss to follow-up after DRE.

This discrepancy may stem from differences in DRE settings; prior studies focused on ophthalmology practices, while ours involved primary care, where point-of-care may reduce structural barriers such as travel and cost by integrating DRE into patients’ routine visits. Additionally, most patients in our cohort had some form of insurance, and the program did not require copayments. Nonetheless, qualitative research indicate that poverty and lack of insurance remain barriers to ongoing ophthalmic care [30,31]. These barriers may be partially mitigated in teleophthalmology-based models, as suggested by prior qualitative work [31], and demonstrated by Bresnick et al., in a study similar to ours [12]. Differences in population characteristics, socioeconomic measures, and study design may therefore explain the lack of association observed in our analysis.

### Clinical factors associated with loss to follow-up

VTDR showed a protective association in bivariate analysis that was not sustained after adjustment. System-level responses, such as prioritization, referral processes, or more intensive patient communication for more severe findings, may explain higher crude adherence among these patients. Previous studies have reported associations between the severity of DR and adherence to follow-up recommendations [12,15,29,32]. Only two studies have explored this association while adjusting for covariates and focusing on POCDREP [12,13]. However with limitations, such as studying a single center or covering mostly an underserved population [12,13], and with the primary care being in the same location as the eye care office [12]. In our analysis, DR severity was not independently associated with loss to follow-up after adjustment.

In contrast, the presence of AMD detected during the DRE was associated with higher odds of loss to follow-up in the multivariable analysis. This finding should be interpreted cautiously, as AMD is strongly correlated with advanced age and frailty [33], which are themselves linked to lower adherence and may reflect underlying barriers rather than a direct causal effect. Older individuals with AMD may face additional challenges, including comorbidities, functional limitations, and logistical constraints, which can further hinder follow-up [26]. Thus, AMD may serve as a marker of patients at higher risk of disengagement from care. Future studies incorporating more detailed geriatric assessments are needed to determine whether this association is independent or reflects underlying vulnerability.

A history of eye disease was associated with lower odds of loss to follow-up. This may reflect greater awareness of the importance of ongoing ophthalmic care or prior engagement with eye care services, which may facilitate adherence to recommended follow-up. Patients with previous eye conditions may better recognize the consequences of delayed care and therefore be more likely to complete follow-up. This interpretation is consistent with findings by Bresnick et al, who reported improved adherence among patients with greater prior interaction with healthcare services, suggesting that familiarity with care pathways may enhance engagement [12].

In contrast, broader measures of comorbidity burden were not independently associated with loss to follow-up after adjustment. Although the number of medical conditions and selected comorbidities showed associations in bivariate analyses, these did not persist in multivariable models. While Bresnick et al. reported that having more than one comorbidity protected against loss to follow-up [12], suggesting that patients with a higher number of medical conditions are accustomed to medical care, explaining their higher adherence [12]. This difference may indicate that, in our cohort, general medical complexity alone was insufficient to drive follow-up adherence once other patient and system-level factors were considered.

Other clinical variables, including smoking status, number of documented medical conditions, mobility-related conditions, diabetes-related complications, were associated with loss to follow-up in unadjusted analyses but not in multivariable models. We did not assess diabetes duration [12] or awareness of glycemic control (HbA1c levels) [8], as these variables were not consistently or accurately documented in the retrospective data. Future research incorporating patient reported measures may help clarify the role of these factors in follow-up adherence.

### System-level factors associated with loss to follow-up

Each percentage-point increase in the overall medical appointment no-show rate was associated with a 4% increase in the odds of loss to follow-up, a finding that remained consistent across all multivariable models. This pattern suggests broader issues, including competing priorities or underlying behavioral tendencies [34,35]. Missed appointments have been linked to younger age, male sex, racial or ethnic backgrounds, low income, homelessness, lower education, and greater distance from healthcare facilities, all of which may contribute to reduced healthcare utilization [34,36]. This association highlights the need for targeted interventions, including personalized reminders, improved accessibility, and patient-centered strategies, to enhance adherence in high-risk individuals [37].

The study results suggest that adherence was influenced by timely patient contact after DRE rather than the method of contact. Shorter intervals between DRE and patient contact were associated with lower odds of loss to follow-up, whereas longer delays were not associated with improved adherence. This aligns with a Rwandan study that found higher follow-up loss with delayed ophthalmologist reports (60.4%) versus immediate AI interpretation (48.5%), with the AI group also attending follow-ups sooner [10]. There has been a gap in the literature regarding the relationship of follow-up with the time from DRE to inform the patient of their results. Our study found an association between patient contact within 30 days of DRE and lower odds of loss to follow up. Reporting results within 30 days was associated with lower odds of loss to follow-up by 63%. This suggests that timely result reporting may prompt patients to prioritize eye care, although the 30-day cutoff should be interpreted as an observed pattern rather than a definitive causal threshold. Additionally, effective communication between primary and eye care providers is crucial for improving follow-up adherence [31,38]. Delays in conveying DRE results may stem from limited ophthalmologic resources, delays in image transfer or interpretation, delayed result retrieval, or gaps in communication between providers, warranting further investigation.

A study in Riverside, CA, found that the likelihood of patients scheduling follow-up appointments in ophthalmological care decreased when the appointment was scheduled at longer interval after testing [12,32]. Patients with recommended intervals of less than 2 weeks and those for 3 months had higher adherence rates compared with those with longer intervals for follow-up [12]. A 2016 study indicated that approximately 1 in 8 patients fail to follow through on referrals from general practitioners to medical specialists [39]. A qualitative study in Tanzania suggested that poor follow-up may stem from unclear referral processes [14]. Patients who exhibit a heightened awareness and sense of ownership towards their condition are more inclined to participate in their care and adhere to follow-up recommendations [40].

In our study, however, documented referral to eye care was not independently associated with follow-up completion. We were unable to assess whether the urgency of recommendations influenced follow-up rates, as this is not standardized in the POCDREP reporting system. This discrepancy may reflect differences in how referrals function within the program, where documentation alone may not capture unmeasured aspects of the referral process, such as the clarity of communication, perceived urgency, or the extent of scheduling support provided to patients. Together, these findings suggest that referral documentation alone may be insufficient to improve follow-up unless referrals are clearly communicated and operationalized within the care pathway.

### Interaction effects

We evaluated multiple interaction terms to assess whether the associations between key predictors and loss to follow-up varied across patient-, clinical-, and system-level factors. Despite testing numerous theoretically plausible interactions, including time from DRE to contact and insurance type, contact method and referral status, and clinical factors such as diabetic complications and BMI, none meaningfully improved model fit based on AIC, BIC, and likelihood ratio testing. Although a small number of interaction terms reached nominal statistical significance, these did not result in improved overall model performance and were therefore not retained (S3 Table).

The absence of meaningful interaction effects should be interpreted with caution. It does not imply that differences across subgroups are unimportant, but rather that we did not detect evidence of effect modification within the variables and sample size available. Overall, these findings suggest that the associations observed in the main models were generally consistent across the subgroups examined.

### Study limitations

As a retrospective study relying on secondary data, our research faces inherent limitations, including potential bias, incomplete information, and missing data. This study may be susceptible to selection bias and residual confounding, because the set of covariates available from the EMR was limited and might not adequately capture all factors influencing the outcome. To mitigate these issues, we implemented standardized data collection procedures, double-reviewed the dependent variable, cross-validated outcomes, and conducted rigorous quality control through data filters and thorough revisions before analysis.

Excluding clinics with limited program implementation has both advantages and limitations. While UPMC data provides a robust dataset, omitting free clinics and FQHCs may restrict generalizability to resource-limited populations. Conversely, including sites with incomplete implementation or inactive in the program could introduce system-level bias, as variability in workflow (e.g., image grading, result reporting, and patient contact) may reflect program performance rather than true determinants of follow-up.

Socioeconomic status was assessed using ZIP code-level poverty quintiles, an ecological proxy and subject to the ecological fallacy. Individual‑level income or education data were not available, and residual confounding by unmeasured individual socioeconomic factors may persist.

Referral processes were standardized and required EMR documentation, though implementation varied across clinics. While this may have led to under-documentation in some cases, our analysis focused on follow-up outcomes rather than referral rates. Additionally, patients who follow up outside the UPMC network may have been misclassified as lost to follow-up. To reduce this risk, clinical teams were previously trained to upload external follow-up reports, and our data review included checks of both chart documentation and the Media section of the medical records. Nonetheless, some outside follow-up encounters may have remained unrecorded.

Finally, findings are specific to a single teleophthalmology program embedded within primary care, which may limit generalizability to other healthcare settings or screening models.

### Future research

Prospective research should evaluate interventions to optimize time-to-contact, including immediate or on-site interpretation through trained non-physician personnel (e.g., nurses, technicians, or lay graders) and AI-assisted systems [41,42], develop targeted strategies for patients with high no-show rates, incorporate patient-reported barriers to care, standardize and operationalize referral workflows, and define optimal management pathways for indeterminate examinations, while assessing scalability and cost-effectiveness across diverse healthcare settings.

## Conclusion

Our study identified key associations with adherence, including patient-level factors (age and marital status), clinical factors (AMD detected on DRE and prior eye disease), and system-level factors (no-show rate and time from DRE to patient contact) associated with loss to follow-up after DRE. Patient contact within 30 days was associated with lower odds of loss to follow-up compared with no recorded contact or contact after 60 days, while a history of eye disease was associated with lower odds of loss to follow-up. While several variables showed modest associations with follow-up adherence, no single factor proved dominant, underscoring the complexity of adherence behavior. Effective improvement may require multifaceted, tailored strategies beyond sociodemographic or socioeconomic profiles. By prioritizing workflow efficiencies that minimize delays and implementing proactive outreach for high-risk individuals, healthcare systems can significantly bridge the gap between DR screening and follow-up for successful treatment, ultimately preventing vision loss.

## Supporting information

S1 Table Factors associated with loss to follow-up after diabetic retinopathy examination in a point-of-care screening program in the US – Model B (Gradable DREs only, n = 2,536). (DOCX)

S2 Table Factors associated with loss to follow-up after diabetic retinopathy examination in a point-of-care screening program in the US – Model C (Indeterminate exams only, n = 400). (DOCX)

S3 Table Interaction term model fit statistics. (DOCX)
