# Supplementary material for: Factors associated with loss to follow-up after point-of-care diabetic retinopathy examination in a teleophthalmology program: A retrospective cohort study
Source: PLoS One. 2026 Oct 29;21(10):e0359305. doi: 10.1371/journal.pone.0359305 (PMC13718922; doi:10.1371/journal.pone.0359305)
Supplement: Supplementary file 1 [file pone.0359305.s001.docx]

**S1 Table. Factors Associated with Loss to Follow-Up After Diabetic Retinopathy Examination in a Point-of-Care Screening Program in the US – Model B (Gradable DREs Only, n=2,536)**

| **Variable** | **Adjusted OR** | **95% CI** | **p-value** |
| --- | --- | --- | --- |
| Age (per year, centered) | 1.016 | 1.002-1.030 | 0.025 |
| Sex |  |  |  |
| Female | (ref) |  |  |
| Male | 0.971 | 0.755-1.250 | 0.821 |
| Race |  |  |  |
| Other | (ref) |  |  |
| Black | 1.079 | 0.580-2.008 | 0.810 |
| White | 1.426 | 0.776-2.619 | 0.253 |
| Marital status |  |  |  |
| Married | (ref) |  |  |
| Not married | 1.416 | 1.071-1.871 | 0.015 |
| Insurance |  |  |  |
| Commercial | (ref) |  |  |
| Dual | 0.712 | 0.436-1.164 | 0.176 |
| Medicaid | 1.067 | 0.714-1.594 | 0.752 |
| Medicare | 0.795 | 0.559-1.130 | 0.201 |
| Veterans Affairs/Uninsured | 0.855 | 0.285-2.563 | 0.779 |
| BMI (kg/m², centered) | 1.011 | 0.995-1.028 | 0.166 |
| Number of medical conditions (centered) | 0.988 | 0.970-1.005 | 0.170 |
| Insulin use |  |  |  |
| No | (ref) |  |  |
| Yes | 0.917 | 0.700-1.203 | 0.533 |
| Smoking status |  |  |  |
| Never smoked | (ref) |  |  |
| Former smoker | 1.152 | 0.872-1.520 | 0.320 |
| Current smoker | 1.314 | 0.930-1.857 | 0.121 |
| Diabetes-related complications |  |  |  |
| No | (ref) |  |  |
| Yes | 0.786 | 0.589-1.049 | 0.102 |
| History of eye disease |  |  |  |
| No | (ref) |  |  |
| Yes | 0.478 | 0.341-0.670 | <0.001 |
| Mobility-related conditions |  |  |  |
| No | (ref) |  |  |
| Yes | 0.877 | 0.663-1.160 | 0.357 |
| VTDR on DRE |  |  |  |
| No | (ref) |  |  |
| Yes | 0.896 | 0.659-1.218 | 0.483 |
| AMD on DRE |  |  |  |
| No | (ref) |  |  |
| Yes | 1.748 | 1.120-2.729 | 0.014 |
| No-show rate (per percentage point, centered) | 1.033 | 1.017-1.048 | <0.001 |
| Referral to eye care |  |  |  |
| No | (ref) |  |  |
| Yes | 0.904 | 0.694-1.178 | 0.456 |
| Time from DRE to patient contact |  |  |  |
| No recorded contact date | (ref) |  |  |
| Same or next day | 0.371 | 0.196-0.702 | 0.002 |
| 2-3 days | 0.476 | 0.251-0.904 | 0.023 |
| 4-5 days | 0.377 | 0.191-0.745 | 0.005 |
| 6-7 days | 0.33 | 0.160-0.680 | 0.003 |
| 8-14 days | 0.408 | 0.206-0.807 | 0.010 |
| 15-30 days | 0.349 | 0.174-0.703 | 0.003 |
| 31-60 days | 0.736 | 0.346-1.565 | 0.426 |
| Contact method |  |  |  |
| No documented contact method | (ref) |  |  |
| Low-tech asynchronous | 1.435 | 0.744-2.766 | 0.282 |
| Phone-based | 0.948 | 0.505-1.779 | 0.869 |
| In-person | 0.971 | 0.494-1.909 | 0.932 |
| Clinic specialty |  |  |  |
| Combined practice (family and internal medicine) | (ref) |  |  |
| Endocrinology | 1.151 | 0.504-2.628 | 0.739 |
| Family Medicine | 1.274 | 0.758-2.139 | 0.360 |
| Internal Medicine | 0.714 | 0.409-1.245 | 0.235 |

***Legend:*** CI: Confidence interval; BMI: Body Mass Index; DRE: Diabetic Retinopathy Examination; VTDR: Vision-threatening diabetic; AMD: Age related Macular Degeneration. Reference categories (ref) are indicated for each variable. Continuous variables were mean-centered.
