# Supplementary material for: Factors associated with loss to follow-up after point-of-care diabetic retinopathy examination in a teleophthalmology program: A retrospective cohort study
Source: PLoS One. 2026 Oct 29;21(10):e0359305. doi: 10.1371/journal.pone.0359305 (PMC13718922; doi:10.1371/journal.pone.0359305)
Supplement: Supplementary file 2 [file pone.0359305.s002.docx]

**S2 Table. Factors Associated with Loss to Follow-Up After Diabetic Retinopathy Examination in a Point-of-Care Screening Program in the US – Model C (Indeterminate Exams Only, n=400)**

| **Variables** | **Adjusted OR** | **95% CI** | **p-value** |
| --- | --- | --- | --- |
| Age (per year, centered) | 1.006 | 0.979-1.033 | 0.686 |
| Sex |  |  |  |
| Female | (ref) |  |  |
| Male | 0.784 | 0.467-1.316 | 0.357 |
| Race |  |  |  |
| Other | (ref) |  |  |
| Black | 1.196 | 0.341-4.197 | 0.780 |
| White | 1.351 | 0.398-4.590 | 0.630 |
| Marital status |  |  |  |
| Married (ref) |  |  |  |
| Not married | 0.96 | 0.569-1.619 | 0.877 |
| Insurance |  |  |  |
| Commercial | (ref) |  |  |
| Dual | 0.75 | 0.268-2.103 | 0.585 |
| Medicaid | 1.525 | 0.526-4.421 | 0.437 |
| Medicare | 0.692 | 0.347-1.379 | 0.295 |
| Veterans Affairs/Uninsured | 0.407 | 0.018-9.153 | 0.571 |
| BMI (kg/m², centered) | 1.032 | 0.995-1.071 | 0.095 |
| Number of medical conditions (centered) | 0.98 | 0.949-1.011 | 0.200 |
| Insulin use |  |  |  |
| No | (ref) |  |  |
| Yes | 0.981 | 0.583-1.650 | 0.941 |
| Smoking status |  |  |  |
| Never smoked | (ref) |  |  |
| Former smoker | 0.757 | 0.432-1.328 | 0.332 |
| Current smoker | 0.756 | 0.369-1.549 | 0.444 |
| Diabetes-related complications |  |  |  |
| No | (ref) |  |  |
| Yes | 1.121 | 0.628-2.001 | 0.700 |
| History of eye disease |  |  |  |
| No | (ref) |  |  |
| Yes | 0.965 | 0.509-1.831 | 0.914 |
| Mobility-related conditions |  |  |  |
| No | (ref) |  |  |
| Yes | 1.344 | 0.785-2.301 | 0.281 |
| No-show rate (per percentage point, centered) | 1.039 | 1.007-1.073 | 0.018 |
| Referral to eye care |  |  |  |
| No | (ref) |  |  |
| Yes | 0.894 | 0.500-1.597 | 0.704 |
| Time from DRE to patient contact |  |  |  |
| Not contacted/>60 days | (ref) |  |  |
| Same or next day | 0.45 | 0.118-1.717 | 0.242 |
| 2-3 days | 0.338 | 0.089-1.284 | 0.111 |
| 4-5 days | 0.866 | 0.212-3.537 | 0.841 |
| 6-7 days | 0.471 | 0.108-2.051 | 0.316 |
| 8-14 days | 0.664 | 0.163-2.705 | 0.567 |
| 15-30 days | 0.806 | 0.175-3.710 | 0.781 |
| 31-60 days | 0.431 | 0.071-2.606 | 0.359 |
| Contact method |  |  |  |
| No documented contact method | (ref) |  |  |
| Low-tech asynchronous | 1.063 | 0.245-4.604 | 0.935 |
| Phone-based | 0.568 | 0.141-2.287 | 0.426 |
| In-person | 1.909 | 0.394-9.256 | 0.422 |
| Clinic specialty |  |  |  |
| Combined practice (family and internal medicine) | (ref) |  |  |
| Endocrinology | 0.429 | 0.101-1.817 | 0.250 |
| Family Medicine | 0.811 | 0.317-2.071 | 0.661 |
| Internal Medicine | 0.746 | 0.254-2.193 | 0.595 |

***Legend:*** CI: Confidence interval; BMI: Body Mass Index; DRE: Diabetic Retinopathy Examination. Model C includes only examinations with indeterminate (ungradable) images; therefore, DRE clinical findings (e.g., VTDR, AMD) and image gradability were not included as predictors. Time from DRE to patient contact is categorized relative to the reference group of not contacted or contact after >60 days. Reference categories (ref) are indicated for each variable. Continuous variables were mean-centered.
