# Supplementary material for: Factors associated with loss to follow-up after point-of-care diabetic retinopathy examination in a teleophthalmology program: A retrospective cohort study
Source: PLoS One. 2026 Oct 29;21(10):e0359305. doi: 10.1371/journal.pone.0359305 (PMC13718922; doi:10.1371/journal.pone.0359305)
Supplement: Supplementary file 3 [file pone.0359305.s003.docx]

**S3 Table. Interaction Term Model Fit Statistics.**

| **Interaction term** | **Log likelihood** | **AIC** | **BIC** | **p-value** |
| --- | --- | --- | --- | --- |
| contact time × insurance type | -1737.717 | 3599.434 | 3970.26 | 0.741 |
| contact time × race | -1745.338 | 3594.676 | 3905.886 | 0.305 |
| contact time × mobility impairment | -1744.754 | 3579.508 | 3848.824 | 0.137 |
| contact time × diabetic complications | -1748.591 | 3587.181 | 3856.497 | 0.111 |
| contact time × clinic specialty | -1741.065 | 3600.13 | 3953.233 | 0.53 |
| contact method × insurance type | -1744.543 | 3589.085 | 3888.326 | 0.218 |
| contact method × race | -1747.127 | 3582.254 | 3845.586 | 0.113 |
| contact method × referral to ophthalmology | -1746.972 | 3575.944 | 3821.321 | 0.052 |
| eye diseases × referral to ophthalmology | -1749.922 | 3577.843 | 3811.25 | 0.04 |
| diabetic complications × BMI | -1749.932 | 3577.865 | 3811.272 | 0.039 |
| clinic specialty × no-show rate | -1748.824 | 3579.648 | 3825.025 | 0.06 |
| contact time × no-show rate | -1748.163 | 3586.326 | 3855.642 | 0.122 |

**Legend**: This table summarizes the model fit statistics for individual mixed-effects logistic regression models including one interaction term at a time. For each tested interaction, the log likelihood, Akaike Information Criterion (AIC), Bayesian Information Criterion (BIC), and Wald test p-value are presented. Lower AIC and BIC values indicate relatively better model fit. Interactions were selected based on theoretical plausibility and prior evidence. Tested interactions included: Time from DRE to patient contact with insurance type, race, mobility impairment, diabetic complications, clinic specialty, and no-show rate; Contact method with insurance type, race, and referral to ophthalmology; Eye diseases with referral to ophthalmology; Diabetic complications with BMI; and Clinic specialty with no-show rate. Most interactions were not statistically significant (all p > 0.05), and none substantially reduced AIC or BIC compared with the main-effects model. Three terms reached nominal significance: eye diseases × referral to ophthalmology (p = 0.040), diabetic complications × BMI (p = 0.039), and contact method × referral to ophthalmology (p = 0.052). Although the in-person contact × referral subgroup showed a large odds ratio (OR = 3.72, 95% CI: 1.20–11.50), the wide confidence interval indicated instability due to sparse data. Given the lack of consistent improvement in model fit, the borderline p-values, and the desire for parsimony and interpretability, no interaction terms were retained in the final model.
