# Supplementary material for: Factors associated with loss to follow-up after point-of-care diabetic retinopathy examination in a teleophthalmology program: A retrospective cohort study
Source: PLoS One. 2026 Oct 29;21(10):e0359305. doi: 10.1371/journal.pone.0359305 (PMC13718922; doi:10.1371/journal.pone.0359305)
Supplement: Supplementary file 5 [file pone.0359305.s006.docx]

**Response to Reviewers**

*Factors associated with loss to follow-up after point-of-care diabetic retinopathy examination in a teleophthalmology program: A retrospective cohort study*

Dear Reviewers,

We thank the reviewers for their careful and constructive comments. We have revised the manuscript accordingly. Our point-by-point responses are provided below.

**Reviewer #1**

**Comment 1:** The authors mentioned in the discussion that some of the factors were not statistically significant in multivariable models but only in bivariate/unadjusted models, indicating confounding. However, confounding should be assessed based on change in estimate between the models rather than by statistical significance. The authors should qualify these sentences.

**Response:** We agree with the reviewer. We revised the Discussion to avoid interpreting a change in statistical significance between bivariate and multivariable analyses as evidence of confounding. Specifically, we removed statements attributing the attenuation or loss of statistical significance after adjustment to confounding and now describe the results directly in terms of the observed unadjusted and adjusted associations.

For VTDR, the previous text stated that attenuation after adjustment “suggests confounding” and that the adjusted findings supported “the role of confounding rather than a direct effect.” These interpretations were removed. The revised manuscript now states:

*“VTDR showed a protective association in bivariate analysis that was not sustained after adjustment.”*

and subsequently:

*“In our analysis, DR severity was not independently associated with loss to follow-up after adjustment.”*

The discussion of possible system-level explanations for the difference between the crude and adjusted findings was retained without characterizing this pattern as confounding.

Similarly, for comorbidity burden, the previous manuscript stated that associations observed in bivariate analyses but not sustained in multivariable models were “suggesting confounding.” This characterization was removed. The revised manuscript now states:

*“Although the number of medical conditions and selected comorbidities showed associations in bivariate analyses, these did not persist in multivariable models.”*

The remaining references to residual confounding occur only in the Study Limitations section, where they appropriately refer to the possibility of unmeasured covariates in this retrospective study rather than to changes in statistical significance between models.

**Reviewer #4**

**Comment:** Line 78: Please clarify sentence, “Sociodemographic and economic factors [associated with higher rates of loss to follow-up]...” as current phrasing is somewhat confusing.

**Response:** We agree and revised the sentence to explicitly state the direction and outcome of the reported associations. The previous text read:

*“Prior studies have identified multiple factors associated with loss to follow-up. Sociodemographic and economic factors include younger age, female sex, urban residence, lack of insurance, and greater distance from eye care services.”*

The revised text now reads:

*“Prior studies have identified several sociodemographic and economic factors associated with loss to follow-up after DRE, including younger age, female sex, urban residence, lack of insurance, and greater distance from eye care services.”*

This revision makes clear that the listed characteristics are factors previously reported to be associated with loss to follow-up after DRE.

**Comment:** Line 109: How did this study verify that once recommended, patients did not seek an eye exam with an office that was outside of the associated POCDREP/EMR? Line 160 states that external eye care encounters were included, but does this only capture external encounters that use the same EMR? Clarification and acknowledgement are needed in the study design section. This is acknowledged in the limitations section Line 709-713 but should be included in study design.

**Response:** We agree and have substantially clarified ascertainment of external eye care encounters in the Methods.

Previously, the manuscript stated only that follow-up included visits within the UPMC Eye Center and documented external eye care encounters. The revised Variables section now specifies that external encounters were counted only when documentation from an outside provider was available within the UPMC EMR. It now states:

*“Follow-up included eye care encounters within the UPMC system as well as external eye care encounters documented in the UPMC EMR. External encounters were identified when documentation from outside providers, such as consultation notes or examination reports, had been entered or uploaded into the patient’s record. The study did not independently query outside health systems or eye care practices; therefore, external encounters that were not documented in the UPMC EMR could not be identified and may have been classified as loss to follow-up.”*

We also expanded the Data Collection Strategy section to describe how these encounters were sought during chart review. The revised manuscript specifies that reviewers searched both for UPMC Eye Center encounters and for documentation of care provided by community or other external eye care providers. For external care, reviewers examined clinical documentation and uploaded records, including the Media section of the EMR. We now explicitly state that external encounters without documentation in the UPMC record could not be captured.

The corresponding limitation was retained in the Study Limitations section. It acknowledges that patients who received follow-up outside the UPMC network may have been misclassified as lost to follow-up if the encounter was not subsequently documented in the UPMC record, despite clinical teams being trained to upload external reports and reviewers checking both chart documentation and the Media section.

We thank the reviewers for these comments, which helped us improve the precision of the statistical interpretation and clarify the ascertainment of follow-up encounters in the manuscript.
