# Supplementary material for: Factors associated with loss to follow-up after point-of-care diabetic retinopathy examination in a teleophthalmology program: A retrospective cohort study
Source: PLoS One. 2026 Oct 29;21(10):e0359305. doi: 10.1371/journal.pone.0359305 (PMC13718922; doi:10.1371/journal.pone.0359305)
Supplement: Supplementary file 6 [file pone.0359305.s005.docx]

Kumar Saurabh

Academic Editor

PlosONE Journal of Ophthalmology Case Reports

Dear Dr. Saurabh, and reviewers

We extend our gratitude for your consideration of our manuscript. Please find attached a revised version originally entitled “Factors associated with loss to follow‑up after point‑of‑care diabetic retinopathy examination in a teleophthalmology program: A retrospective cohort study” Manuscript Number: PONE-D-25-66892

We highly value the comments and suggestions provided by the reviewers. Their insights have been instrumental in refining the quality of our manuscript, which has undergone thorough editing. We trust that the revisions incorporated in the manuscript, along with our accompanying responses, will meet the standards required for publication.

Thank you for your attention to our work.

With best regards,

**Francisco J. Bonilla-Escobar, MD, MSc, PhD**

**Journal Requirements:**

When submitting your revision, we need you to address these additional requirements.

1. Please ensure that your manuscript meets PLOS ONE's style requirements, including those for f**ile naming.** The PLOS ONE style templates can be found at

[https://journals.plos.org/plosone/s/file?id=wjVg/PLOSOne_formatting_sample_main_body.pdf](https://track.editorialmanager.com/CL0/https:%2F%2Fjournals.plos.org%2Fplosone%2Fs%2Ffile%3Fid=wjVg%2FPLOSOne_formatting_sample_main_body.pdf/1/010f019d476cfc5a-24e7d101-4e5b-4c0c-a7a9-2992a7ea33f4-000000/Ts804OSywRtd0IrDLjz--qxI4ExTmV9P3zIEfcwu24Y=257) and

[https://journals.plos.org/plosone/s/file?id=ba62/PLOSOne_formatting_sample_title_authors_affiliations.pdf](https://track.editorialmanager.com/CL0/https:%2F%2Fjournals.plos.org%2Fplosone%2Fs%2Ffile%3Fid=ba62%2FPLOSOne_formatting_sample_title_authors_affiliations.pdf/1/010f019d476cfc5a-24e7d101-4e5b-4c0c-a7a9-2992a7ea33f4-000000/vioXOWglKO4k8xT0-k_R_SghrsEbIZley3wqJVJnOfY=257)

**Response**: Thank you for the comment, we have done as suggested and adhered to the guidelines according to the templates, furthermore we renamed our files in the platform and within the manuscript.

2. For studies involving **third-party data,** we encourage authors to share any **data specific to their analyses that they can legally distribute.** PLOS recognizes, however, that authors may be using third-party data they do not have the rights to share. When third-party data cannot be publicly shared, authors must provide **all information necessary for interested** researchers to apply **to gain access to the data.** (<https://journals.plos.org/plosone/s/data-availability#loc-acceptable-data-access-restrictions>)

For any third-party data that the authors cannot legally distribute, they should include the following information in their **Data Availability Statement upon submission:**

1) A description of the data set and the third-party source

2) If applicable, verification of permission to use the data set

3) Confirmation of whether the **authors received any special privileges i**n accessing the data that other researchers would not have

4) **All necessary contact information others would need to apply to gain access to the** data

**Response**:

Thank you for this important comment. The dataset used in this study was derived from third-party institutional electronic medical record and program data from the University of Pittsburgh/UPMC Point-of-Care Diabetic Retinopathy Examination Program. Because these data contain protected health information and are not owned by the authors for public redistribution, the raw dataset cannot be made publicly available. In accordance with PLOS policy, we will revise the Data Availability Statement to clarify the nature and source of the dataset, confirm that the data were accessed under University of Pittsburgh Institutional Review Board approval (STUDY19120237), and state that the authors did not receive special access privileges beyond those available to other qualified researchers. We will also include the appropriate contact for data access requests. Qualified researchers may request access to the data through the program leadership, subject to institutional review and data use agreements.

Data Availability Statement:

“The data underlying this study are not publicly available due to restrictions imposed by the University of Pittsburgh Institutional Review Board and UPMC data governance policies, as the dataset contains potentially identifiable health information.

Data may be made available to qualified researchers upon reasonable request and with appropriate institutional approvals. Requests for access should be directed to the UPMC Clinical Data Warehouse (CDW), which manages clinical data extraction and governance for UPMC:

UPMC Clinical Data Warehouse

Email: cdwhelp@upmc.edu

Website: https://www.upmc.com/services/data-analytics/cdw

In addition, requests may require review and approval by the University of Pittsburgh Institutional Review Board (IRB), which oversees human subjects research compliance:

University of Pittsburgh IRB

Email: irb@pitt.edu

Website: https://www.irb.pitt.edu

Researchers requesting access will need to submit a proposal and obtain appropriate ethical and institutional approvals prior to data release.”

3. We note that you have included the phrase **“data not shown”** in your manuscript. Unfortunately, this does not meet our **data sharing requirements.** PLOS does not permit references to inaccessible data. We require that authors provide all relevant data within the paper, Supporting Information files, or in an acceptable, public repository. Please add a citation to support this phrase or upload the data that corresponds with these findings to a stable repository (such as Figshare or Dryad) and provide and URLs, DOIs, or accession numbers that may be used to access these data. Or, if the data are not a core part of the research being presented in your study, we ask that you **remove the phrase that refers to these data.**

**Response**: Thank you for the comment, we have done as suggested, and removed the Data not shown, since these data are not a core part of the research being presented in our study.

4. If the reviewer comments include a recommendation to cite specific previously published works, please review and evaluate these publications to determine whether they are relevant and should be cited. There is no requirement to cite these works unless the editor has indicated otherwise.

**Response**: Thank you for the comment, we have done as suggested.

Reviewers' comments:

**Review Comments to the Author**

Please use the space provided to explain your answers to the questions above. You may also include additional comments for the author, including concerns about dual publication, research ethics, or publication ethics. (Please upload your review as an attachment if it exceeds 20,000 characters)

**Reviewer #1:** Minor points:
1) In the abstract results, it should be **"a higher no-show rate"** rather than "A higher no-show rate"

**Response**: Thank you for the comment, we have done as suggested.

2) In the abstract results, 95% CI were presented inconsistently: 95% CI: vs. 95% CI=

**Response**: Thank you for the comment, we have done as suggested. Standardized to “95% CI = ” throughout the abstract and manuscript.

3) In the abstract results, "were associated with protective factor, preventing loss to follow-up," could be simplified to "were associated with lower loss to follow-up,"

**Response**: Thank you for the comment, we have done as suggested.

4) In bivariate analysis and multivariable analysis results', the percentages were inconsistently presented: sometimes providing the contrasting groups (x% vs. y%), while sometimes only for a group (e.g., AMD (x%, P=...)

**Response**: We appreciate the reviewer’s observation regarding the presentation of these variables. As these data points represent dichotomous categories, they are presented in a singular format to specifically emphasize the levels that reached statistical significance. We believe this approach prioritizes the most clinically relevant findings while maintaining concise data reporting. Furthermore the complete data can be seen in the table.

Major points:
5) The outcome was loss to follow-up after a DRE within 6 months. However, it was not clear that the follow-up was also recommended within 6 months. Authors excluded those who had an exam within a year after the initial DRE with the same results. However, it is not clear that for some DREs, even if the results were of higher severity, they were still in recommended window of period, just not within 6 months. Authors should clearly state that the recommendations for follow-up should be within 6 months, otherwise those DREs would be treated as loss to follow-up.

**Response**: Thank you for the comment. We would like to clarify that, within our teleophthalmology program, follow-up is not assigned based on fixed time intervals (e.g., 6 months). Instead, all patients with a positive screening result—defined as more than mild diabetic retinopathy, presence of diabetic macular edema, incidental findings suggestive of other relevant ocular pathology, or ungradable images—are recommended to seek evaluation by an eye care specialist.

Regarding outcome definition, we excluded subsequent exams that yielded the same findings as the index exam, as these do not represent true clinical follow-up with an eye care provider but rather repeat screening encounters. In line with the reviewer’s concern, such cases were classified as loss to follow-up, since appropriate referral to eye care was not completed.

We have added a clear explanation in the Methods – Participants section: “Within our teleophthalmology program, follow‑up is not assigned based on fixed time intervals. All patients with a positive screening result (more than mild DR, macular edema, incidental pathology, or ungradable images) are recommended for eye care evaluation. Repeat exams within a year with identical findings were excluded because they do not represent true clinical follow‑up; these were classified as loss to follow‑up because appropriate referral was not completed.”

6) In bivariate results, even though some factors did not achieve statistical significance (p<=0.05), they have borderline/trending significance (p<0.10 or even p<0.15) and the effect sizes were comparable to those factors who had statistical significance. These should be acknowledged.

**Response**: We appreciate the reviewer’s feedback. While we have incorporated a clarifying statement regarding this point, we have deliberately avoided the use of terms such as ‘borderline’ or ‘near significance.’ To maintain strict statistical rigor and adhere to a predefined alpha level of 0.05, we have reported our findings based solely on whether they met the threshold for 95% CI. We believe this approach ensures a more conservative and robust interpretation of the results.

7) Multivariable model C was performed on n=400 ungradable photos, and not many factors were significant. This could be due to smaller sample size, and should be acknowledged.

**Response**: Thank you for the comment, we have added a comment on this in the interaction effects section in the discussion.

8) The major finding of the study is that the follow-up adherence seems to be multi-faceted (clinical, sociodemographic/economic, and system-level factors) and it was emphasized in many instances throughout the manuscript. However, in discussion, when referring to interaction models being not significant, it was suggested that tailored/personalized approach is not needed but a generalized or a broad approach is. Authors should address this to be consistent.

**Response**: We thank the reviewer for highlighting this apparent inconsistency. We agree that our findings emphasize the multifactorial nature of follow-up adherence, involving clinical, sociodemographic, and system-level factors. The statement regarding the lack of significant interaction effects was not intended to suggest that a generalized (one-size-fits-all) approach is universally optimal, nor to dismiss the value of tailored interventions. Rather, we sought to convey a more specific interpretation: the absence of significant interactions between key predictors (e.g., contact timing, method of communication) and patient-level characteristics (e.g., insurance type, race, mobility impairment) suggests that certain system-level interventions—particularly timely result reporting—may exert broadly protective effects that transcend many individual risk factors.

In other words, while patient-level factors such as age, marital status, and comorbidity burden independently influence adherence, the effectiveness of reducing communication delays does not appear to vary significantly across the subgroups we tested. This supports the implementation of universal workflow improvements (e.g., ensuring result reporting within 30 days) as a foundational strategy that benefits most patients, without negating the need for additional, targeted support for higher-risk populations (e.g., older unmarried individuals, those with AMD, or patients with high no-show rates).

9) In study limitations, authors should acknowledge bias, specifically, potential selection bias and confounding due to the nature of retrospective cohort study design and limited recorded factors which might not be sufficient for addressing confounding.

**Response:** Thank you for the comment, we have added a phrase in the limitation section addressing this concern: “*this study may be susceptible to selection bias and residual confounding, because the set of covariates available from the electronic medical records was limited and might not adequately capture all factors influencing the outcome.”*

**Reviewer #2:** This manuscript addresses an important and clinically relevant issue—loss to follow-up after diabetic retinopathy (DR) tele-screening. The relatively large dataset and inclusion of system-level variables are strengths. However, there are several methodological and interpretational concerns that need to be addressed before the findings can be considered robust.
Major Comments
1. There is an inconsistency in the reporting of the study population. The abstract indicates that 3,121 DREs were obtained from 2,959 patients, suggesting repeated measures for some individuals, whereas the Methods section describes a final sample of 3,121 patients. This discrepancy requires clarification. If multiple examinations per patient were included, the analysis should account for within-patient correlation. Please clearly define the unit of analysis and ensure consistency throughout the manuscript.

**Response:** Thank you for this important observation.Thank you for this important comment. We agree that the description of the study population and unit of analysis required clarification. The dataset included 3,121 diabetic retinopathy exams obtained from 2,959 unique patients, as some individuals contributed more than one exam during the study period.

For all analyses, the unit of analysis was the exam (N=3,121). To appropriately account for repeated measurements within individuals, we incorporated clustering at the patient level in all relevant analyses, thereby addressing within-patient correlation.

We acknowledge that this was not clearly stated in the original version. We have revised the Abstract, Methods, and Tables to consistently distinguish between number of exams and number of patients, explicitly define the unit of analysis, and describe the use of patient-level clustering in the statistical approach. In addition, we have updated the title of Table 1,2 and 3 to reflect the exam-level unit of analysis, now reading: “Bivariate Comparison of Sociodemographic and Economic Characteristics by Follow-Up Status Among Diabetic Retinopathy Exams Included in the Analysis (N=3,121 Exams from 2,959 Patients).”

2. Age: The reported effect size is small (aOR ~1.02 per year), yet it is interpreted as a major determinant.
AMD: The association with loss to follow-up may be confounded by age and comorbidity burden.

**Response:** Thank you for the comment, We agree that the interpretation of age and AMD findings requires tempering. We have revised the manuscript as follows:

Age: We now describe age as a “modest but statistically significant predictor” rather than a “major determinant,” while noting that small per‑year effects accumulate over decades. We also acknowledge that age may be a proxy for unmeasured barriers (e.g., mobility, cognitive decline).

AMD: We now explicitly acknowledge that residual confounding by age and comorbidity burden cannot be excluded, and we have added a limitation statement to that effect. We also clarify that the association should be interpreted cautiously.

3. Terminology - Use consistent terminology: “loss to follow-up” vs “non-follow-up”

**Response:** We have standardized the term to “loss to follow‑up” (or “lost to follow‑up”) throughout the manuscript.

4. Ensure all figures are self-explanatory and consistent with tables

**Response:** Thank you for the comment, we have revised Figure 1 legend to explicitly state the reference category and the outcome (loss to follow‑up) which is consistent with table 4. Figure 2 legend now defines all abbreviations (DRE, etc.). The figures´ title and legend are embedded in the manuscript as submission guidelines indicate.

5. Clarify definition of “follow-up” (e.g., any visit vs condition-specific visit)

**Response:** Thank you for the comment, we have added a clear definition in the Methods section (Variables subsection) : *“Follow‑up was defined as attendance at any appointment with an eye care provider (optometrist or ophthalmologist) within 180 days after the index DRE, regardless of whether the visit specifically addressed diabetic retinopathy or other ocular conditions.”*

6. The sample size calculation: Appears unnecessary in a retrospective cohort including all eligible patients
Uses assumptions that are not clearly justified

**Response:** Thank you for the comment, we agree. The sample size calculation was carried out prospectively during study planning but is not needed for a retrospective analysis of the full cohort. We have removed the “Sample Size” subsection entirely to avoid confusion.

7. Socioeconomic Status - Based on ZIP code-level poverty, which is a weak proxy and prone to ecological fallacy.

**Response**: We acknowledge this limitation. We have added a clear statement in the Limitations section: “Socioeconomic status was assessed using ZIP code‑level poverty quintiles, an ecological proxy subject to the ecological fallacy. Individual‑level income or education data were not available.”

**Reviewer #3:** Abstract:

Lines 33-35: It was not the electronic medical records that were analyzed directly; rather, follow-up details were extracted from the EMR, and loss-to-follow-up outcomes were analyzed using clustered multivariate logistic regression to assess socioeconomic, clinical, and system-level factors.

**Response:** Thank you for the comment, we have clarified this in the line you mention.

Line 65: Is it the prevalence of diabetes or DR

**Response:** Thank you for the comment,this is the prevalence of DR, we have clarified this adding *“...affecting approximately 9.6 million individuals* ***with diabetes*** *in the United States, of whom 1.84 million have vision‑threatening diabetic retinopathy (VTDR).”*

Line 68: POCDREP can be described in short and introduced suddenly in the second paragraph.

**Response:** Thank you for the comment, We acknowledge the reviewer’s point. However, we elected to maintain the current structure, as the first paragraph was already densely packed with several key concepts; separating the content enhanced clarity and readability.

Line 106: ophthalmologist or optometrist

**Response:** Thank you for the comment, we have done as suggested.

Line 103-110 : Be consistent with reporting dates; remove "st" next to dates or be consistent.

**Response:** Thank you for the comment, we have done as suggested.

Line 120-123: The POCDREP at the University of Pittsburgh/UPMC included 35 primary care offices equipped with automated non-mydriatic retinal cameras, serving patients referred from a total of 75 offices. Patients from the remaining 40 offices without cameras were referred to one of the 35 equipped locations for DRE.
Line can be rewritten as " A total of 75 centers were involved, including 35 equipped with automated cameras and 40 without cameras. Patients from centers without cameras were referred to centers where cameras were available for screening.

**Response:** Thank you for the comment, we have done as suggested.

Line 131: Between two ophthalmologists, is the grading process standardized or not? Please mention it.

**Response:** Thank you for the comment, we added a sentence to clarify this: “…*and reported by two practicing ophthalmologists using a standardized grading protocol based on the International Clinical Diabetic Retinopathy severity scale. Disagreements were resolved by consensus.”*

Line 141 : Repeated exams within a year that yielded the same results (n=369). can you rewrite to make it clear " Patients attending multiple times with the same screening results"

**Response:** Thank you for the comment we have done as suggested.

Line 185: expand PCP

**Response:** Thank you for the comment we have done as suggested.

Line 192-193: with patients assigned to the most resource-intensive method. Please explain this.

**Response:** Thank you for the comment, we added a sentence to clarify this: *For example, if a patient was both called and emailed, the contact method was classified as ‘phone‑based’ (the more resource‑intensive modality)*

Line 213-214: The search was conducted twice by different members of the research team to ensure accuracy in the revision. This sentence can end with accuracy. Revision is confusing.
**Response:** Thank you for the comment, we have done as suggested.

Line 218: POCDRED, change it to POCDREP

**Response:** Thank you for the comment, we have done as suggested.

Line 218: remove (2)

**Response:** Thank you for the comment, we have done as suggested.

Line 288: Rewrite the sentence, as it is confusing. Consider this: "A total of 3,121 diabetic retinopathy examinations (DREs) from 2,959 unique patients who were recommended for ophthalmic follow-up were included in the analysis."

Response: Thank you for the comment, we have done as suggested.

Line 297: Can rework the sentence. Can be written as "Most patients had type 2 diabetes (91.4%), and 42.1% were using insulin."

**Response:** Thank you for the comment, we have done as suggested.

Lines 329-330: Start the new sentence with "Patients who attended follow-up had a higher median number of documented medical conditions than those who did not (13 vs. 11; p < 0.001)."
**Response:** Thank you for the comment, we have done as suggested.

Line 332: The linked word can be replaced with "associated."

**Response:** Thank you for the comment, we have done as suggested.

Line 360: replace photo with image acquisition

**Response:** Thank you for the comment, we have done as suggested.

Line 361: Replace "referral to ophthalmology" with "ophthalmologist or optometrist."

**Response:** Thank you for the comment, we have done as suggested.

Line 382: Shorter interval between DRE exam to contact

**Response:** Thank you for the comment, we have done as suggested.

Line 451-452: Delayed result reporting independently reduced follow-up likelihood... Can be written as "Delayed test results reporting..."

**Response:** Thank you for the comment, we have done as suggested.

Lines 473-476: It is good to corroborate your study findings with previously reported data, with age , gender, etc.

**Response:** Thank you for the comment, we have done as suggested. We have revised multiple studies that encounter different data and contrasted with our results. *“Other studies have described younger age,(8, 10) sex (female), site of residence,(10) distance to the eye centers,(11) and Latino ethnicity,(12, 13) as associated factors with lost to follow-up. We adjusted for most variables except distance and ethnicity; location data was identifiable, and prior studies showing ethnic associations were biased toward Latino populations, as they comprised 65% and 72% Latinos,(12, 13) which exceeded the actual Latino population in their respective study areas (Riverside, CA: 52% Latino; Durham, NC: 13.9% Latino). In contrast, our study reflects the true Latino representation in our region (2.5%),(22) limiting our ability to assess associations due to the small sample size….*

*…Older age also remained a modest but statistically significant predictor of loss to follow-up, through multiples models of multivariate analyses, linking …”*

Line 512-515: Can you further clarify why poverty is not associated with loss of follow-up in this study? You attributed it to primary care in your study. Can you further explain, like, since primary care happens in their vicinity or it is free services, etc.?

**Response:** Thank you for the comment, we added a sentence to clarify this: “...*This discrepancy may stem from differences in DRE settings; prior studies focused on ophthalmology practices, while ours involved primary care patients, which reduces travel and financial barriers by offering DREs within patients’ usual care settings. Additionally, the majority of patients had some form of insurance, and the program did not require copayments for the screening itself.”*

Using the different models (A, B and C) for multiple logistic regression analyses, this approach is methodologically rigorous when subgroups differ clinically. Also improves internal validity of the model. Can you highlight across three models which predictors are significant?

Appropriate statistical tests were used, including testing the regression models' fit.

**Response:** Thank you for this helpful comment. We agree that, while presenting separate models (A, B, and C) is methodologically appropriate given the clinical differences between subgroups, the manuscript would benefit from a clearer synthesis of significant predictors across models. In response, we have revised the Results section to explicitly highlight which variables were consistently associated with loss to follow-up across models and which were model-specific. We have also added a summary description to facilitate comparison of findings across Models A, B, and C.

Added a synthesis paragraph in Results – Multivariable analysis: *“Across models A and B, older age, not being married, and higher no‑show rates were associated with higher odds of loss to follow‑up, whereas only no‑show rate remained significant in Model C. A history of eye disease was protective in Models A and B. AMD remained significant in Models A and B but was not assessable in Model C. Shorter DRE‑to‑contact intervals were associated with lower odds in Models A and B, with similar patterns in Model C.”*

In general, a few sentences are confusing for readers. Authors may check on these and correct them in the revision.

**Response:** We thank the reviewer for this general comment. We have carefully reviewed the entire manuscript and identified several sentences that could be clarified. These have been revised as indicated in the point‑by‑point responses above
