# Supplementary material for: Factors associated with loss to follow-up after point-of-care diabetic retinopathy examination in a teleophthalmology program: A retrospective cohort study
Source: PLoS One. 2026 Oct 29;21(10):e0359305. doi: 10.1371/journal.pone.0359305 (PMC13718922; doi:10.1371/journal.pone.0359305)
Supplement: Peer review history

# Peer review: Decision Letter 0

**DOI:** 10.1371/journal.pone.0359305.r001

**Authors:** Kumar Saurabh

1 Apr 2026

Dear Dr. Bonilla-Escobar,

Thank you for submitting your manuscript to PLOS ONE. After careful consideration, we feel that it has merit but does not fully meet PLOS ONE’s publication criteria as it currently stands. Therefore, we invite you to submit a revised version of the manuscript that addresses the points raised during the review process.

Please submit your revised manuscript by May 16 2026 11:59PM. If you will need more time than this to complete your revisions, please reply to this message or contact the journal office at plosone@plos.org. When you're ready to submit your revision, log on to https://www.editorialmanager.com/pone/ and select the 'Submissions Needing Revision' folder to locate your manuscript file.

- A letter that responds to each point raised by the academic editor and reviewer(s). You should upload this letter as a separate file labeled 'Response to Reviewers'.
- A marked-up copy of your manuscript that highlights changes made to the original version. You should upload this as a separate file labeled 'Revised Manuscript with Track Changes'.
- An unmarked version of your revised paper without tracked changes. You should upload this as a separate file labeled 'Manuscript'.

If you would like to make changes to your financial disclosure, please include your updated statement in your cover letter. Guidelines for resubmitting your figure files are available below the reviewer comments at the end of this letter.

If applicable, we recommend that you deposit your laboratory protocols in protocols.io to enhance the reproducibility of your results. Protocols.io assigns your protocol its own identifier (DOI) so that it can be cited independently in the future. For instructions see: https://journals.plos.org/plosone/s/submission-guidelines#loc-laboratory-protocols. Additionally, PLOS ONE offers an option for publishing peer-reviewed Lab Protocol articles, which describe protocols hosted on protocols.io. Read more information on sharing protocols at https://plos.org/protocols?utm_medium=editorial-email&utm_source=authorletters&utm_campaign=protocols.

As the corresponding author, your ORCID iD is verified in the submission system and will appear in the published article. PLOS supports the use of ORCID, and we encourage all coauthors to register for an ORCID iD and use it as well. Please encourage your coauthors to verify their ORCID iD within the submission system before final acceptance, as unverified ORCID iDs will not appear in the published article. *Only*  the individual author can complete the verification step; PLOS staff *cannot*  verify ORCID iDs on behalf of authors.

We look forward to receiving your revised manuscript.

Kind regards,

Kumar Saurabh

Academic Editor

PLOS One

**Journal Requirements:**

When submitting your revision, we need you to address these additional requirements.

1. Please ensure that your manuscript meets PLOS ONE's style requirements, including those for file naming. The PLOS ONE style templates can be found at

https://journals.plos.org/plosone/s/file?id=wjVg/PLOSOne_formatting_sample_main_body.pdf and

https://journals.plos.org/plosone/s/file?id=ba62/PLOSOne_formatting_sample_title_authors_affiliations.pdf

2. For studies involving third-party data, we encourage authors to share any data specific to their analyses that they can legally distribute. PLOS recognizes, however, that authors may be using third-party data they do not have the rights to share. When third-party data cannot be publicly shared, authors must provide all information necessary for interested researchers to apply to gain access to the data. (https://journals.plos.org/plosone/s/data-availability#loc-acceptable-data-access-restrictions)

For any third-party data that the authors cannot legally distribute, they should include the following information in their Data Availability Statement upon submission:

1) A description of the data set and the third-party source

2) If applicable, verification of permission to use the data set

3) Confirmation of whether the authors received any special privileges in accessing the data that other researchers would not have

4) All necessary contact information others would need to apply to gain access to the data

3. We note that you have included the phrase “data not shown” in your manuscript. Unfortunately, this does not meet our data sharing requirements. PLOS does not permit references to inaccessible data. We require that authors provide all relevant data within the paper, Supporting Information files, or in an acceptable, public repository. Please add a citation to support this phrase or upload the data that corresponds with these findings to a stable repository (such as Figshare or Dryad) and provide and URLs, DOIs, or accession numbers that may be used to access these data. Or, if the data are not a core part of the research being presented in your study, we ask that you remove the phrase that refers to these data.

4. If the reviewer comments include a recommendation to cite specific previously published works, please review and evaluate these publications to determine whether they are relevant and should be cited. There is no requirement to cite these works unless the editor has indicated otherwise.

[Note: HTML markup is below. Please do not edit.]

Reviewers' comments:

Reviewer's Responses to Questions

**Comments to the Author**

1. Is the manuscript technically sound, and do the data support the conclusions?

Reviewer #1: Yes

Reviewer #2: Partly

Reviewer #3: Yes

**********

2. Has the statistical analysis been performed appropriately and rigorously?

Reviewer #1: Yes

Reviewer #2: No

Reviewer #3: Yes

**********

3. Have the authors made all data underlying the findings in their manuscript fully available?

The PLOS Data policy

Reviewer #1: Yes

Reviewer #2: No

Reviewer #3: Yes

**********

4. Is the manuscript presented in an intelligible fashion and written in standard English?

Reviewer #1: Yes

Reviewer #2: Yes

Reviewer #3: No

**********

Reviewer #1: Minor points:

1) In the abstract results, it should be "a higher no-show rate" rather than "A higher no-show rate"

2) In the abstract results, 95% CI were presented inconsistently: 95% CI: vs. 95% CI=

3) In the abstract results, "were associated with protective factor, preventing loss to follow-up," could be simplified to "were associated with lower loss to follow-up,"

4) In bivariate analysis and multivariable analysis results', the percentages were inconsistently presented: sometimes providing the contrasting groups (x% vs. y%), while sometimes only for a group (e.g., AMD (x%, P=...)

Major points:

5) The outcome was loss to follow-up after a DRE within 6 months. However, it was not clear that the follow-up was also recommended within 6 months. Authors excluded those who had an exam within a year after the initial DRE with the same results. However, it is not clear that for some DREs, even if the results were of higher severity, they were still in recommended window of period, just not within 6 months. Authors should clearly state that the recommendations for follow-up should be within 6 months, otherwise those DREs would be treated as loss to follow-up.

6) In bivariate results, even though some factors did not achieve statistical significance (p<=0.05), they have borderline/trending significance (p<0.10 or even p<0.15) and the effect sizes were comparable to those factors who had statistical significance. These should be acknowledged.

7) Multivariable model C was performed on n=400 ungradable photos, and not many factors were significant. This could be due to smaller sample size, and should be acknowledged.

8) The major finding of the study is that the follow-up adherence seems to be multi-faceted (clinical, sociodemographic/economic, and system-level factors) and it was emphasized in many instances throughout the manuscript. However, in discussion, when referring to interaction models being not significant, it was suggested that tailored/personalized approach is not needed but a generalized or a broad approach is. Authors should address this to be consistent.

9) In study limitations, authors should acknowledge bias, specifically, potential selection bias and confounding due to the nature of retrospective cohort study design and limited recorded factors which might not be sufficient for addressing confounding.

Reviewer #2: This manuscript addresses an important and clinically relevant issue—loss to follow-up after diabetic retinopathy (DR) tele-screening. The relatively large dataset and inclusion of system-level variables are strengths. However, there are several methodological and interpretational concerns that need to be addressed before the findings can be considered robust.

Major Comments

1. There is an inconsistency in the reporting of the study population. The abstract indicates that 3,121 DREs were obtained from 2,959 patients, suggesting repeated measures for some individuals, whereas the Methods section describes a final sample of 3,121 patients. This discrepancy requires clarification. If multiple examinations per patient were included, the analysis should account for within-patient correlation. Please clearly define the unit of analysis and ensure consistency throughout the manuscript.

2. Age: The reported effect size is small (aOR ~1.02 per year), yet it is interpreted as a major determinant.

AMD: The association with loss to follow-up may be confounded by age and comorbidity burden.

3. Terminology - Use consistent terminology: “loss to follow-up” vs “non-follow-up”

4. Ensure all figures are self-explanatory and consistent with tables

5. Clarify definition of “follow-up” (e.g., any visit vs condition-specific visit)

6. The sample size calculation: Appears unnecessary in a retrospective cohort including all eligible patients

Uses assumptions that are not clearly justified

7. Socioeconomic Status - Based on ZIP code-level poverty, which is a weak proxy and prone to ecological fallacy.

Reviewer #3: Abstract:

Lines 33-35: It was not the electronic medical records that were analyzed directly; rather, follow-up details were extracted from the EMR, and loss-to-follow-up outcomes were analyzed using clustered multivariate logistic regression to assess socioeconomic, clinical, and system-level factors.

Line 65: Is it the prevalence of diabetes or DR

Line 68: POCDREP can be described in short and introduced suddenly in the second paragraph.

Line 106: ophthalmologist or optometrist

Line 103-110 : Be consistent with reporting dates; remove "st" next to dates or be consistent.

Line 120-123: The POCDREP at the University of Pittsburgh/UPMC included 35 primary care offices equipped with automated non-mydriatic retinal cameras, serving patients referred from a total of 75 offices. Patients from the remaining 40 offices without cameras were referred to one of the 35 equipped locations for DRE.

Line can be rewritten as " A total of 75 centers were involved, including 35 equipped with automated cameras and 40 without cameras. Patients from centers without cameras were referred to centers where cameras were available for screening.

Line 131: Between two ophthalmologists, is the grading process standardized or not? Please mention it.

Line 141 : Repeated exams within a year that yielded the same results (n=369). can you rewrite to make it clear " Patients attending multiple times

with the same screening results"

Line 185: expand PCP

Line 192-193: with patients assigned to the most resource-intensive method. Please explain this.

Line 213-214: The search was conducted twice by different members of the research team to ensure accuracy in the revision. This sentence can end with accuracy. Revision is confusing.

Line 218: POCDRED, change it to POCDREP

Line 218: remove (2)

Line 288: Rewrite the sentence, as it is confusing. Consider this: "A total of 3,121 diabetic retinopathy examinations (DREs) from 2,959 unique patients who were recommended for ophthalmic follow-up were included in the analysis."

Line 297: Can rework the sentence. Can be written as "Most patients had type 2 diabetes (91.4%), and 42.1% were using insulin."

Lines 329-330: Start the new sentence with "Patients who attended follow-up had a higher median number of documented medical conditions than those who did not (13 vs. 11; p < 0.001)."

Line 332: The linked word can be replaced with "associated."

Line 360: replace photo with image acquisition

Line 361: Replace "referral to ophthalmology" with "ophthalmologist or optometrist."

Line 382: Shorter interval between DRE exam to contact

Line 451-452: Delayed result reporting independently reduced follow-up likelihood... Can be written as "Delayed test results reporting..."

Lines 473-476: It is good to corroborate your study findings with previously reported data, with age , gender, etc.

Line 512-515: Can you further clarify why poverty is not associated with loss of follow-up in this study? You attributed it to primary care in your study. Can you further explain, like, since primary care happens in their vicinity or it is free services, etc.?

Using the different models (A, B and C) for multiple logistic regression analyses, this approach is methodologically rigorous when subgroups differ clinically. Also improves internal validity of the model. Can you highlight across three models which predictors are significant?

Appropriate statistical tests were used, including testing the regression models' fit.

In general, a few sentences are confusing for readers. Authors may check on these and correct them in the revision.

**********

what does this mean?). If published, this will include your full peer review and any attached files.

If you choose “no”, your identity will remain anonymous but your review may still be made public.

**Do you want your identity to be public for this peer review?**  For information about this choice, including consent withdrawal, please see our Privacy Policy

Reviewer #1: No

Reviewer #2: **Yes:**  Aswin PR

Reviewer #3: No

**********

[NOTE: If reviewer comments were submitted as an attachment file, they will be attached to this email and accessible via the submission site. Please log into your account, locate the manuscript record, and check for the action link "View Attachments". If this link does not appear, there are no attachment files.]

To ensure your figures meet our technical requirements, please review our figure guidelines: https://journals.plos.org/plosone/s/figures You may also use PLOS’s free figure tool, NAAS, to help you prepare publication quality figures: https://journals.plos.org/plosone/s/figures#loc-tools-for-figure-preparation.

NAAS will assess whether your figures meet our technical requirements by comparing each figure against our figure specifications.

# Author comment: Author response to Decision Letter 1

**DOI:** 10.1371/journal.pone.0359305.r002

28 Apr 2026

Kumar Saurabh

Academic Editor

PlosONE Journal of Ophthalmology Case Reports

Dear Dr. Saurabh, and reviewers

We extend our gratitude for your consideration of our manuscript. Please find attached a revised version originally entitled “Factors associated with loss to follow‑up after point‑of‑care diabetic retinopathy examination in a teleophthalmology program: A retrospective cohort study” Manuscript Number: PONE-D-25-66892

We highly value the comments and suggestions provided by the reviewers. Their insights have been instrumental in refining the quality of our manuscript, which has undergone thorough editing. We trust that the revisions incorporated in the manuscript, along with our accompanying responses, will meet the standards required for publication.

Thank you for your attention to our work.

With best regards,

Francisco J. Bonilla-Escobar, MD, MSc, PhD

Journal Requirements:

When submitting your revision, we need you to address these additional requirements.

1. Please ensure that your manuscript meets PLOS ONE's style requirements, including those for file naming. The PLOS ONE style templates can be found at

https://journals.plos.org/plosone/s/file?id=wjVg/PLOSOne_formatting_sample_main_body.pdf and

https://journals.plos.org/plosone/s/file?id=ba62/PLOSOne_formatting_sample_title_authors_affiliations.pdf

Response: Thank you for the comment, we have done as suggested and adhered to the guidelines according to the templates, furthermore we renamed our files in the platform and within the manuscript.

2. For studies involving third-party data, we encourage authors to share any data specific to their analyses that they can legally distribute. PLOS recognizes, however, that authors may be using third-party data they do not have the rights to share. When third-party data cannot be publicly shared, authors must provide all information necessary for interested researchers to apply to gain access to the data. (https://journals.plos.org/plosone/s/data-availability#loc-acceptable-data-access-restrictions)

For any third-party data that the authors cannot legally distribute, they should include the following information in their Data Availability Statement upon submission:

1) A description of the data set and the third-party source

2) If applicable, verification of permission to use the data set

3) Confirmation of whether the authors received any special privileges in accessing the data that other researchers would not have

4) All necessary contact information others would need to apply to gain access to the data

Response:

Thank you for this important comment. The dataset used in this study was derived from third-party institutional electronic medical record and program data from the University of Pittsburgh/UPMC Point-of-Care Diabetic Retinopathy Examination Program. Because these data contain protected health information and are not owned by the authors for public redistribution, the raw dataset cannot be made publicly available. In accordance with PLOS policy, we will revise the Data Availability Statement to clarify the nature and source of the dataset, confirm that the data were accessed under University of Pittsburgh Institutional Review Board approval (STUDY19120237), and state that the authors did not receive special access privileges beyond those available to other qualified researchers. We will also include the appropriate contact for data access requests. Qualified researchers may request access to the data through the program leadership, subject to institutional review and data use agreements.

Data Availability Statement:

“The data underlying this study are not publicly available due to restrictions imposed by the University of Pittsburgh Institutional Review Board and UPMC data governance policies, as the dataset contains potentially identifiable health information.

Data may be made available to qualified researchers upon reasonable request and with appropriate institutional approvals. Requests for access should be directed to the UPMC Clinical Data Warehouse (CDW), which manages clinical data extraction and governance for UPMC:

UPMC Clinical Data Warehouse

Email: cdwhelp@upmc.edu

Website: https://www.upmc.com/services/data-analytics/cdw⁠

In addition, requests may require review and approval by the University of Pittsburgh Institutional Review Board (IRB), which oversees human subjects research compliance:

University of Pittsburgh IRB

Email: irb@pitt.edu

Website: https://www.irb.pitt.edu⁠

Researchers requesting access will need to submit a proposal and obtain appropriate ethical and institutional approvals prior to data release.”

3. We note that you have included the phrase “data not shown” in your manuscript. Unfortunately, this does not meet our data sharing requirements. PLOS does not permit references to inaccessible data. We require that authors provide all relevant data within the paper, Supporting Information files, or in an acceptable, public repository. Please add a citation to support this phrase or upload the data that corresponds with these findings to a stable repository (such as Figshare or Dryad) and provide and URLs, DOIs, or accession numbers that may be used to access these data. Or, if the data are not a core part of the research being presented in your study, we ask that you remove the phrase that refers to these data.

Response: Thank you for the comment, we have done as suggested, and removed the Data not shown, since these data are not a core part of the research being presented in our study.

4. If the reviewer comments include a recommendation to cite specific previously published works, please review and evaluate these publications to determine whether they are relevant and should be cited. There is no requirement to cite these works unless the editor has indicated otherwise.

Response: Thank you for the comment, we have done as suggested.

Reviewers' comments:

Review Comments to the Author

Please use the space provided to explain your answers to the questions above. You may also include additional comments for the author, including concerns about dual publication, research ethics, or publication ethics. (Please upload your review as an attachment if it exceeds 20,000 characters)

Reviewer #1: Minor points:

1) In the abstract results, it should be "a higher no-show rate" rather than "A higher no-show rate"

Response: Thank you for the comment, we have done as suggested.

2) In the abstract results, 95% CI were presented inconsistently: 95% CI: vs. 95% CI=

Response: Thank you for the comment, we have done as suggested. Standardized to “95% CI = ” throughout the abstract and manuscript.

3) In the abstract results, "were associated with protective factor, preventing loss to follow-up," could be simplified to "were associated with lower loss to follow-up,"

Response: Thank you for the comment, we have done as suggested.

4) In bivariate analysis and multivariable analysis results', the percentages were inconsistently presented: sometimes providing the contrasting groups (x% vs. y%), while sometimes only for a group (e.g., AMD (x%, P=...)

Response: We appreciate the reviewer’s observation regarding the presentation of these variables. As these data points represent dichotomous categories, they are presented in a singular format to specifically emphasize the levels that reached statistical significance. We believe this approach prioritizes the most clinically relevant findings while maintaining concise data reporting. Furthermore the complete data can be seen in the table.

Major points:

5) The outcome was loss to follow-up after a DRE within 6 months. However, it was not clear that the follow-up was also recommended within 6 months. Authors excluded those who had an exam within a year after the initial DRE with the same results. However, it is not clear that for some DREs, even if the results were of higher severity, they were still in recommended window of period, just not within 6 months. Authors should clearly state that the recommendations for follow-up should be within 6 months, otherwise those DREs would be treated as loss to follow-up.

Response: Thank you for the comment. We would like to clarify that, within our teleophthalmology program, follow-up is not assigned based on fixed time intervals (e.g., 6 months). Instead, all patients with a positive screening result—defined as more than mild diabetic retinopathy, presence of diabetic macular edema, incidental findings suggestive of other relevant ocular pathology, or ungradable images—are recommended to seek evaluation by an eye care specialist.

Regarding outcome definition, we excluded subsequent exams that yielded the same findings as the index exam, as these do not represent true clinical follow-up with an eye care provider but rather repeat screening encounters. In line with the reviewer’s concern, such cases were classified as loss to follow-up, since appropriate referral to eye care was not completed.

We have added a clear explanation in the Methods – Participants section: “Within our teleophthalmology program, follow‑up is not assigned based on fixed time intervals. All patients with a positive screening result (more than mild DR, macular edema, incidental pathology, or ungradable images) are recommended for eye care evaluation. Repeat exams within a year with identical findings were excluded because they do not represent true clinical follow‑up; these were classified as loss to follow‑up because appropriate referral was not completed.”

6) In bivariate results, even though some factors did not achieve statistical significance (p<=0.05), they have borderline/trending significance (p<0.10 or even p<0.15) and the effect sizes were comparable to those factors who had statistical significance. These should be acknowledged.

Response: We appreciate the reviewer’s feedback. While we have incorporated a clarifying statement regarding this point, we have deliberately avoided the use of terms such as ‘borderline’ or ‘near significance.’ To maintain strict statistical rigor and adhere to a predefined alpha level of 0.05, we have reported our findings based solely on whether they met the threshold for 95% CI. We believe this approach ensures a more conservative and robust interpretation of the results.

7) Multivariable model C was performed on n=400 ungradable photos, and not many factors were significant. This could be due to smaller sample size, and should be acknowledged.

Response: Thank you for the comment, we have added a comment on this in the interaction effects section in the discussion.

8) The major finding of the study is that the follow-up adherence seems to be multi-faceted (clinical, sociodemographic/economic, and system-level factors) and it was emphasized in many instances throughout the manuscript. However, in discussion, when referring to interaction models being not significant, it was suggested that tailored/personalized approach is not needed but a generalized or a broad approach is. Authors should address this to be consistent.

Response: We thank the reviewer for highlighting this apparent inconsistency. We agree that our findings emphasize the multifactorial nature of follow-up adherence, involving clinical, sociodemographic, and system-level factors. The statement regarding the lack of significant interaction effects was not intended to suggest that a generalized (one-size-fits-all) approach is universally optimal, nor to dismiss the value of tailored interventions. Rather, we sought to convey a more specific interpretation: the absence of significant interactions between key predictors (e.g., contact timing, method of communication) and patient-level characteristics (e.g., insurance type, race, mobility impairment) suggests that certain system-level interventions—particularly timely result reporting—may exert broadly protective effects that transcend many individual risk factors.

In other words, while patient-level factors such as age, marital status, and comorbidity burden independently influence adherence, the effectiveness of reducing communication delays does not appear to vary significantly across the subgroups we tested. This supports the implementation of universal workflow improvements (e.g., ensuring result reporting within 30 days) as a foundational strategy that benefits most patients, without negating the need for additional, targeted support for higher-risk populations (e.g., older unmarried individuals, those with AMD, or patients with high no-show rates).

9) In study limitations, authors should acknowledge bias, specifically, potential selection bias and confounding due to the nature of retrospective cohort study design and limited recorded factors which might not be sufficient for addressing confounding.

Response: Thank you for the comment, we have added a phrase in the limitation section addressing this concern: “this study may be susceptible to selection bias and residual confounding, because the set of covariates available from the electronic medical records was limited and might not adequately capture all factors influencing the outcome.”

Reviewer #2: This manuscript addresses an important and clinically relevant issue—loss to follow-up after diabetic retinopathy (DR) tele-screening. The relatively large dataset and inclusion of system-level variables are strengths. However, there are several methodological and interpretational concerns that need to be addressed before the findings can be considered robust.

Major Comments

1. There is an inconsistency in the reporting of the study population. The abstract indicates that 3,121 DREs were obtained from 2,959 patients, suggesting repeated measures for some individuals, whereas the Methods section describes a final sample of 3,121 patients. This discrepancy requires clarification. If multiple examinations per patient were included, the analysis should account for within-patient correlation. Please clearly define the unit of analysis and ensure consistency throughout the manuscript.

Response: Thank you for this important observation.Thank you for this important comment. We agree that the description of the study population and unit of analysis required clarification. The dataset included 3,121 diabetic retinopathy exams obtained from 2,959 unique patients, as some individuals contributed more than one exam during the study period.

For all analyses, the unit of analysis was the exam (N=3,121). To appropriately account for repeated measurements within individuals, we incorporated clustering at the patient level in all relevant analyses, thereby addressing within-patient correlation.

We acknowledge that this was not clearly stated in the original version. We have revised the Abstract, Methods, and Tables to consistently distinguish between number of exams and number of patients, explicitly define the unit of analysis, and describe the use of patient-level clustering in the statistical approach. In addition, we have updated the title of Table 1,2 and 3 to reflect the exam-level unit of analysis, now reading: “Bivariate Comparison of Sociodemographic and Economic Characteristics by Follow-Up Status Among Diabetic Retinopathy Exams Included in the Analysis (N=3,121 Exams from 2,959 Patients).”

2. Age: The reported effect size is small (aOR ~1.02 per year), yet it is interpreted as a major determinant.

AMD: The association with loss to follow-up may be confounded by age and comorbidity burden.

Response: Thank you for the comment, We agree that the interpretation of age and AMD findings requires tempering. We have revised the manuscript as follows:

Age: We now describe age as a “modest but statistically significant predictor” rather than a “major determinant,” while noting that small per‑year effects accumulate over decades. We also acknowledge that age may be a proxy for unmeasured barriers (e.g., mobility, cognitive decline).

AMD: We now explicitly acknowledge that residual confounding by age and comorbidity burden cannot be excluded, and we have added a limitation statement to that effect. We also clarify that the association should be interpreted cautiously.

3. Terminology - Use consistent terminology: “loss to follow-up” vs “non-follow-up”

Response: We have standardized the term to “loss to follow‑up” (or “lost to follow‑up”) throughout the manuscript.

4. Ensure all figures are self-explanatory and consistent with tables

Response: Thank you for the comment, we have revised Figure 1 legend to explicitly state the reference category and the outcome (loss to follow‑up) which is consistent with table 4. Figure 2 legend now defines all abbreviations (DRE, etc.). The figures´ title and legend are embedded in the manuscript as submission guidelines indicate.

5. Clarify definition of “follow-up” (e.g., any visit vs condition-specific visit)

Response: Thank you for the comment, we have added a clear definition in the Methods section (Variables subsection) : “Follow‑up was defined as attendance at any appointment with an eye care provider (optometrist or ophthalmologist) within 180 days after the index DRE, regardless of whether the visit specifically addressed diabetic retinopathy or other ocular conditions.”

6. The sample size calculation: Appears unnecessary in a retrospective cohort including all eligible patients

Uses assumptions that are not clearly justified

Response: Thank you for the comment, we agree. The sample size calculation was carried out prospectively during study planning but is not needed for a retrospective analysis of the full cohort. We have removed the “Sample Size” subsection entirely to avoid confusion.

7. Socioeconomic Status - Based on ZIP code-level poverty, which is a weak proxy and prone to ecological fallacy.

Response: We acknowledge this limitation. We have added a clear statement in the Limitations section: “Socioeconomic status was assessed using ZIP code‑level poverty quintiles, an ecological proxy subject to the ecological fallacy. Individual‑level income or education data were not available.”

Reviewer #3: Abstract:

Lines 33-35: It was not the electronic medical records that were analyzed directly; rather, follow-up details were extracted from the EMR, and loss-to-follow-up outcomes were analyzed using clustered multivariate logistic regression to assess socioeconomic, clinical, and system-level factors.

Response: Thank you for the comment, we have clarified this in the line you mention.

Line 65: Is it the prevalence of diabetes or DR

Response: Thank you for the comment,this is the prevalence of DR, we have clarified this adding “...affecting approximately 9.6 million individuals with diabetes in the United States, of whom 1.84 million have vision‑threatening diabetic retinopathy (VTDR).”

Line 68: POCDREP can be described in short and introduced suddenly in the second paragraph.

Response: Thank you for the comment, We acknowledge the reviewer’s point. However, we elected to maintain the current structure, as the first paragraph was already densely packed with several key concepts; separating the content enhanced clarity and readability.

Line 106: ophthalmologist or optometrist

Response: Thank you for the comment, we have done as suggested.

Line 103-110 : Be consistent with reporting dates; remove "st" next to dates or be consistent.

Response: Thank you for the comment, we have done as suggested.

Line 120-123: The POCDREP at the University of Pittsburgh/UPMC included 35 primary care offices equipped with automated non-mydriatic retinal cameras, serving patients referred from a total of 75 offices. Patients from the remaining 40 offices without cameras were referred to one of the 35 equipped locations for DRE.

Line can be rewritten as " A total of 75 centers were involved, including 35 equipped with automated cameras and 40 without cameras. Patients from centers without cameras were referred to centers where cameras were available for screening.

Response: Thank you for the comment, we have done as suggested.

Line 131: Between two ophthalmologists, is the grading process standardized or not? Please mention it.

Response: Thank you for the comment, we added a sentence to clarify this: “…and reported by two practicing ophthalmologists using a standardized grading protocol based on the International Clinical Diabetic Retinopathy severity scale. Disagreements were resolved by consensus.”

Line 141 : Repeated exams within a year that yielded the same results (n=369). can you rewrite to make it clear " Patients attending multiple times with the same screening results"

Response: Thank you for the comment we have done as suggested.

Line 185: expand PCP

Response: Thank you for the comment we have done as suggested.

Line 192-193: with patients assigned to the most resource-intensive method. Please explain this.

Response: Thank you for the comment, we added a sentence to clarify this: For example, if a patient was both called and emailed, the contact method was classified as ‘phone‑based’ (the more resource‑intensive modality)

Line 213-214: The search was conducted twice by different members of the research team to ensure accuracy in the revision. This sentence can end with accuracy. Revision is confusing.

Response: Thank you for the comment, we have done as suggested.

Line 218: POCDRED, change it to POCDREP

Response: Thank you for the comment, we have done as suggested.

Line 218: remove (2)

Response: Thank you for the comment, we have done as suggested.

Line 288: Rewrite the sentence, as it is confusing. Consider this: "A total of 3,121 diabetic retinopathy examinations (DREs) from 2,959 unique patients who were recommended for ophthalmic follow-up were included in the analysis."

Response: Thank you for the comment, we have done as suggested.

Line 297: Can rework the sentence. Can be written as "Most patients had type 2 diabetes (91.4%), and 42.1% were using insulin."

Response: Thank you for the comment, we have done as suggested.

Lines 329-330: Start the new sentence with "Patients who attended follow-up had a higher median number of documented medical conditions than those who did not (13 vs. 11; p < 0.001)."

Response: Thank you for the comment, we have done as suggested.

Line 332: The linked word can be replaced with "associated."

Response: Thank you for the comment, we have done as suggested.

Line 360: replace photo with image acquisition

Response: Thank you for the comment, we have done as suggested.

Line 361: Replace "referral to ophthalmology" with "ophthalmologist or optometrist."

Response: Thank you for the comment, we have done as suggested.

Line 382: Shorter interval between DRE exam to contact

Response: Thank you for the comment, we have done as suggested.

Line 451-452: Delayed result reporting independently reduced follow-up likelihood... Can be written as "Delayed test results reporting..."

Response: Thank you for the comment, we have done as suggested.

Lines 473-476: It is good to corroborate your study findings with previously reported data, with age , gender, etc.

Response: Thank you for the comment, we have done as suggested. We have revised multiple studies that encounter different data and contrasted with our results. “Other studies have described younger age,(8, 10) sex (female), site of residence,(10) distance to the eye centers,(11) and Latino ethnicity,(12, 13) as associated factors with lost to follow-up. We adjusted for most variables except distance and ethnicity; location data was identifiable, and prior studies showing ethnic associations were biased toward Latino populations, as they comprised 65% and 72% Latinos,(12, 13) which exceeded the actual Latino population in their respective study areas (Riverside, CA: 52% Latino; Durham, NC: 13.9% Latino). In contrast, our study reflects the true Latino representation in our region (2.5%),(22) limiting our ability to assess associations due to the small sample size….

…Older age also remained a modest but statistically significant predictor of loss to follow-up, through multiples models of multivariate analyses, linking …”

Line 512-515: Can you further clarify why poverty is not associated with loss of follow-up in this study? You attributed it to primary care in your study. Can you further explain, like, since primary care happens in their vicinity or it is free services, etc.?

Response: Thank you for the comment, we added a sentence to clarify this: “...This discrepancy may stem from differences in DRE settings; prior studies focused on ophthalmology practices, while ours involved primary care patients, which reduces travel and financial barriers by offering DREs within patients’ usual care settings. Additionally, the majority of patients had some form of insurance, and the program did not require copayments for the screening itself.”

Using the different models (A, B and C) for multiple logistic regression analyses, this approach is methodologically rigorous when subgroups differ clinically. Also improves internal validity of the model. Can you highlight across three models which predictors are significant?

Appropriate statistical tests were used, including testing the regression models' fit.

Response: Thank you for this helpful comment. We agree that, while presenting separate models (A, B, and C) is methodologically appropriate given the clinical differences between subgroups, the manuscript would benefit from a clearer synthesis of significant predictors across models. In response, we have revised the Results section to explicitly highlight which variables were consistently associated with loss to follow-up across models and which were model-specific. We have also added a summary description to facilitate comparison of findings across Models A, B, and C.

Added a synthesis paragraph in Results – Multivariable analysis: “Across models A and B, older age, not being married, and higher no‑show rates were associated with higher odds of loss to follow‑up, whereas only no‑show rate remained significant in Model C. A history of eye disease was protective in Models A and B. AMD remained significant in Models A and B but was not assessable in Model C. Shorter DRE‑to‑contact intervals were associated with lower odds in Models A and B, with similar patterns in Model C.”

In general, a few sentences are confusing for readers. Authors may check on these and correct them in the revision.

Response: We thank the reviewer for this general comment. We have carefully reviewed the entire manuscript and identified several sentences that could be clarified. These have been revised as indicated in the point‑by‑point responses above

________________________________________

Attachment Submitted filename: Response to Reviewers Pone.docx

# Peer review: Decision Letter 1

**DOI:** 10.1371/journal.pone.0359305.r003

**Authors:** Shree Kurup

3 Sep 2026

Dear Dr. Bonilla-Escobar,

Thank you for submitting your manuscript to PLOS One. After careful consideration, we feel that it has merit but does not fully meet PLOS One’s publication criteria as it currently stands. Therefore, we invite you to submit a revised version of the manuscript that addresses the points raised during the review process.

Please submit your revised manuscript by Oct 18 2026 11:59PM. If you will need more time than this to complete your revisions, please reply to this message or contact the journal office at plosone@plos.org. When you're ready to submit your revision, log on to https://www.editorialmanager.com/pone/ and select the 'Submissions Needing Revision' folder to locate your manuscript file.

- A letter that responds to each point raised by the academic editor and reviewer(s). You should upload this letter as a separate file labeled 'Response to Reviewers'.
- A marked-up copy of your manuscript that highlights changes made to the original version. You should upload this as a separate file labeled 'Revised Manuscript with Track Changes'.
- An unmarked version of your revised paper without tracked changes. You should upload this as a separate file labeled 'Manuscript'.

If you would like to make changes to your financial disclosure, please include your updated statement in your cover letter. Guidelines for resubmitting your figure files are available below the reviewer comments at the end of this letter.

If applicable, we recommend that you deposit your laboratory protocols in protocols.io to enhance the reproducibility of your results. Protocols.io assigns your protocol its own identifier (DOI) so that it can be cited independently in the future. For instructions see: https://journals.plos.org/plosone/s/submission-guidelines#loc-laboratory-protocols. Additionally, PLOS One offers an option for publishing peer-reviewed Lab Protocol articles, which describe protocols hosted on protocols.io. Read more information on sharing protocols at https://plos.org/protocols?utm_medium=editorial-email&utm_source=authorletters&utm_campaign=protocols.

As the corresponding author, your ORCID iD is verified in the submission system and will appear in the published article. PLOS supports the use of ORCID, and we encourage all coauthors to register for an ORCID iD and use it as well. Please encourage your coauthors to verify their ORCID iD within the submission system before final acceptance, as unverified ORCID iDs will not appear in the published article. *Only*  the individual author can complete the verification step; PLOS staff *cannot*  verify ORCID iDs on behalf of authors.

We look forward to receiving your revised manuscript.

Kind regards,

Shree K. Kurup, MD

Academic Editor

PLOS One

Journal Requirements:

If the reviewer comments include a recommendation to cite specific previously published works, please review and evaluate these publications to determine whether they are relevant and should be cited. There is no requirement to cite these works unless the editor has indicated otherwise.

Please review your reference list to ensure that it is complete and correct. If you have cited papers that have been retracted, please include the rationale for doing so in the manuscript text, or remove these references and replace them with relevant current references. Any changes to the reference list should be mentioned in the rebuttal letter that accompanies your revised manuscript. If you need to cite a retracted article, indicate the article’s retracted status in the References list and also include a citation and full reference for the retraction notice.

Additional Editor Comments:

This journal is happy to receive your manuscript after

minor revisions. Please read the reviews comments very carefully

[Note: HTML markup is below. Please do not edit.]

Reviewers' comments:

Reviewer's Responses to Questions

**Comments to the Author**

Reviewer #1: All comments have been addressed

Reviewer #4: (No Response)

**********

2. Is the manuscript technically sound, and do the data support the conclusions?

Reviewer #1: Yes

Reviewer #4: Yes

**********

3. Has the statistical analysis been performed appropriately and rigorously?

Reviewer #1: Yes

Reviewer #4: Yes

**********

4. Have the authors made all data underlying the findings in their manuscript fully available?

The PLOS Data policy

Reviewer #1: Yes

Reviewer #4: No

**********

5. Is the manuscript presented in an intelligible fashion and written in standard English?

Reviewer #1: Yes

Reviewer #4: Yes

**********

Reviewer #1: 1. The authors mentioned in the discussion that some of the factors were not statistically significant in multivariable models but only in bivariate/unadjusted models, indicating confounding. However, confounding should be assessed based on change in estimate between the models rather than by statistical significance. The authors should qualify these sentences.

Reviewer #4: Line 78: Please clarify sentence, "Sociodemographic and economic factors [associated with higher rates of loss to follow-up]..." as current phrasing is somewhat confusing

Line 109: How did this study verify that once recommended, patients did not seek an eye exam with an office that was outside of the associated POCDREP/EMR? Line 160 states that external eye care encounters were included, but does this only capture external encounters that use the same EMR? Clarification and acknowledgement are needed in the study design section. This is acknowledged in the limitations section Line 709-713 but should be included in study design.

**********

what does this mean?). If published, this will include your full peer review and any attached files.

If you choose “no”, your identity will remain anonymous but your review may still be made public.

**Do you want your identity to be public for this peer review?**  For information about this choice, including consent withdrawal, please see our Privacy Policy

Reviewer #1: No

Reviewer #4: No

**********

[NOTE: If reviewer comments were submitted as an attachment file, they will be attached to this email and accessible via the submission site. Please log into your account, locate the manuscript record, and check for the action link "View Attachments". If this link does not appear, there are no attachment files.]

To ensure your figures meet our technical requirements, please review our figure guidelines: https://journals.plos.org/plosone/s/figures

You may also use PLOS’s free figure tool, NAAS, to help you prepare publication quality figures: https://journals.plos.org/plosone/s/figures#loc-tools-for-figure-preparation.

NAAS will assess whether your figures meet our technical requirements by comparing each figure against our figure specifications.

# Author comment: Author response to Decision Letter 2

**DOI:** 10.1371/journal.pone.0359305.r004

5 Sep 2026

Dear Reviewers,

We thank the reviewers for their careful and constructive comments. We have revised the manuscript accordingly. Our point-by-point responses are provided below.

Reviewer #1

Comment 1: The authors mentioned in the discussion that some of the factors were not statistically significant in multivariable models but only in bivariate/unadjusted models, indicating confounding. However, confounding should be assessed based on change in estimate between the models rather than by statistical significance. The authors should qualify these sentences.

Response: We agree with the reviewer. We revised the Discussion to avoid interpreting a change in statistical significance between bivariate and multivariable analyses as evidence of confounding. Specifically, we removed statements attributing the attenuation or loss of statistical significance after adjustment to confounding and now describe the results directly in terms of the observed unadjusted and adjusted associations.

For VTDR, the previous text stated that attenuation after adjustment “suggests confounding” and that the adjusted findings supported “the role of confounding rather than a direct effect.” These interpretations were removed. The revised manuscript now states:

“VTDR showed a protective association in bivariate analysis that was not sustained after adjustment.”

and subsequently:

“In our analysis, DR severity was not independently associated with loss to follow-up after adjustment.”

The discussion of possible system-level explanations for the difference between the crude and adjusted findings was retained without characterizing this pattern as confounding.

Similarly, for comorbidity burden, the previous manuscript stated that associations observed in bivariate analyses but not sustained in multivariable models were “suggesting confounding.” This characterization was removed. The revised manuscript now states:

“Although the number of medical conditions and selected comorbidities showed associations in bivariate analyses, these did not persist in multivariable models.”

The remaining references to residual confounding occur only in the Study Limitations section, where they appropriately refer to the possibility of unmeasured covariates in this retrospective study rather than to changes in statistical significance between models.

Reviewer #4

Comment: Line 78: Please clarify sentence, “Sociodemographic and economic factors [associated with higher rates of loss to follow-up]...” as current phrasing is somewhat confusing.

Response: We agree and revised the sentence to explicitly state the direction and outcome of the reported associations. The previous text read:

“Prior studies have identified multiple factors associated with loss to follow-up. Sociodemographic and economic factors include younger age, female sex, urban residence, lack of insurance, and greater distance from eye care services.”

The revised text now reads:

“Prior studies have identified several sociodemographic and economic factors associated with loss to follow-up after DRE, including younger age, female sex, urban residence, lack of insurance, and greater distance from eye care services.”

This revision makes clear that the listed characteristics are factors previously reported to be associated with loss to follow-up after DRE.

Comment: Line 109: How did this study verify that once recommended, patients did not seek an eye exam with an office that was outside of the associated POCDREP/EMR? Line 160 states that external eye care encounters were included, but does this only capture external encounters that use the same EMR? Clarification and acknowledgement are needed in the study design section. This is acknowledged in the limitations section Line 709-713 but should be included in study design.

Response: We agree and have substantially clarified ascertainment of external eye care encounters in the Methods.

Previously, the manuscript stated only that follow-up included visits within the UPMC Eye Center and documented external eye care encounters. The revised Variables section now specifies that external encounters were counted only when documentation from an outside provider was available within the UPMC EMR. It now states:

“Follow-up included eye care encounters within the UPMC system as well as external eye care encounters documented in the UPMC EMR. External encounters were identified when documentation from outside providers, such as consultation notes or examination reports, had been entered or uploaded into the patient’s record. The study did not independently query outside health systems or eye care practices; therefore, external encounters that were not documented in the UPMC EMR could not be identified and may have been classified as loss to follow-up.”

We also expanded the Data Collection Strategy section to describe how these encounters were sought during chart review. The revised manuscript specifies that reviewers searched both for UPMC Eye Center encounters and for documentation of care provided by community or other external eye care providers. For external care, reviewers examined clinical documentation and uploaded records, including the Media section of the EMR. We now explicitly state that external encounters without documentation in the UPMC record could not be captured.

The corresponding limitation was retained in the Study Limitations section. It acknowledges that patients who received follow-up outside the UPMC network may have been misclassified as lost to follow-up if the encounter was not subsequently documented in the UPMC record, despite clinical teams being trained to upload external reports and reviewers checking both chart documentation and the Media section.

We thank the reviewers for these comments, which helped us improve the precision of the statistical interpretation and clarify the ascertainment of follow-up encounters in the manuscript.

Attachment Submitted filename: Response_to_Reviewers_POCDREP.docx

# Editor report: Decision Letter 2

**DOI:** 10.1371/journal.pone.0359305.r005

**Authors:** Shree Kurup

14 Sep 2026

Factors associated with loss to follow-up after point-of-care diabetic retinopathy examination in a teleophthalmology program: A retrospective cohort study

PONE-D-25-66892R2

Dear Dr. Bonilla-Escobar,

We’re pleased to inform you that your manuscript has been judged scientifically suitable for publication and will be formally accepted for publication once it meets all outstanding technical requirements.

Within one week, you’ll receive an e-mail detailing the required amendments. When these have been addressed, you’ll receive a formal acceptance letter and your manuscript will be scheduled for publication.

An invoice will be generated when your article is formally accepted. Please note, if your institution has a publishing partnership with PLOS and your article meets the relevant criteria, all or part of your publication costs will be covered. Please make sure your user information is up-to-date by logging into Editorial Manager at Editorial Manager® and clicking the ‘Update My Information' link at the top of the page. For questions related to billing, please contact billing support.

If your institution or institutions have a press office, please notify them about your upcoming paper to help maximize its impact. If they’ll be preparing press materials, please inform our press team as soon as possible -- no later than 48 hours after receiving the formal acceptance. Your manuscript will remain under strict press embargo until 2 pm Eastern Time on the date of publication. For more information, please contact onepress@plos.org.

Kind regards,

Shree K. Kurup, MD

Academic Editor

PLOS One

Additional Editor Comments (optional):

Reviewers' comments:

# Editor report: Acceptance letter

**DOI:** 10.1371/journal.pone.0359305.r006

**Authors:** Shree Kurup

PONE-D-25-66892R2

PLOS One

Dear Dr. Bonilla-Escobar,

I'm pleased to inform you that your manuscript has been deemed suitable for publication in PLOS One. Congratulations! Your manuscript is now being handed over to our production team.

At this stage, our production department will prepare your paper for publication. This includes ensuring the following:

* All references, tables, and figures are properly cited

* All relevant supporting information is included in the manuscript submission,

* There are no issues that prevent the paper from being properly typeset

You will receive further instructions from the production team, including instructions on how to review your proof when it is ready. Please keep in mind that we are working through a large volume of accepted articles, so please give us a few days to review your paper and let you know the next and final steps.

Lastly, if your institution or institutions have a press office, please let them know about your upcoming paper now to help maximize its impact. If they'll be preparing press materials, please inform our press team within the next 48 hours. Your manuscript will remain under strict press embargo until 2 pm Eastern Time on the date of publication. For more information, please contact onepress@plos.org.

You will receive an invoice from PLOS for your publication fee after your manuscript has reached the completed accept phase. If you receive an email requesting payment before acceptance or for any other service, this may be a phishing scheme. Learn how to identify phishing emails and protect your accounts at https://explore.plos.org/phishing.

If we can help with anything else, please email us at customercare@plos.org.

Thank you for submitting your work to PLOS One and supporting open access.

Kind regards,

PLOS One Editorial Office Staff

on behalf of

Dr. Shree K. Kurup

Academic Editor

PLOS One
